# Sensilla Morphology and Complex Expression Pattern of Odorant Binding Proteins in the Vetch Aphid *Megoura viciae* (Hemiptera: Aphididae)

**DOI:** 10.3389/fphys.2018.00777

**Published:** 2018-06-25

**Authors:** Daniele Bruno, Gerarda Grossi, Rosanna Salvia, Andrea Scala, Donatella Farina, Annalisa Grimaldi, Jing-Jiang Zhou, Sabino A. Bufo, Heiko Vogel, Ewald Grosse-Wilde, Bill S. Hansson, Patrizia Falabella

**Affiliations:** ^1^Department of Biotechnology and Life Sciences, University of Insubria, Varese, Italy; ^2^Department of Sciences, University of Basilicata, Potenza, Italy; ^3^Department of Biological Chemistry and Crop Protection, Rothamsted Research, Harpenden, United Kingdom; ^4^Department of Entomology, Max Planck Institute for Chemical Ecology, Jena, Germany; ^5^Department of Evolutionary Neuroethology, Max Planck Institute for Chemical Ecology, Jena, Germany

**Keywords:** vetch aphid, chemoreception, odorant-binding proteins, RT-qPCR, immunolocalization, behavioral assays

## Abstract

Chemoreception in insects is mediated by several components interacting at different levels and including odorant-binding proteins (OBPs). Although recent studies demonstrate that the function of OBPs cannot be restricted to an exclusively olfactory role, and that OBPs have been found also in organs generally not related to chemoreception, their feature of binding molecules remains undisputed. Studying the vetch aphid *Megoura viciae* (Buckton), we used a transcriptomic approach to identify ten OBPs in the antennae and we examined the ultrastructural morphology of sensilla and their distribution on the antennae, legs, mouthparts and cauda of wingless and winged adults by scanning electron microscopy (SEM). Three types of sensilla, trichoid, coeloconic and placoid, differently localized and distributed on antennae, mouthparts, legs and cauda, were described. The expression analysis of the ten OBPs was performed by RT-qPCR in the antennae and other body parts of the wingless adults and at different developmental stages and morphs. Five of the ten OBPs (*MvicOBP1, MvicOBP3, MvicOBP6, MvicOBP7*, and *MvicOBP8*), whose antibodies were already available, were selected for experiments of whole-mount immunolocalization on antennae, mouthparts, cornicles and cauda of adult aphids. Most of the ten OBPs were more expressed in antennae than in other body parts; *MvicOBP1, MvicOBP3, MvicOBP6, MvicOBP7* were also immunolocalized in the sensilla on the antennae, suggesting a possible involvement of these proteins in chemoreception. *MvicOBP6, MvicOBP7, MvicOBP8, MvicOBP9* were highly expressed in the heads and three of them (*MvicOBP6, MvicOBP7, MvicOBP8*) were immunolocalized in the sensilla on the mouthparts, supporting the hypothesis that also mouthparts may be involved in chemoreception. *MvicOBP2, MvicOBP3, MvicOBP5, MvicOBP8* were highly expressed in the cornicles-cauda and two of them (*MvicOBP3, MvicOBP8)* were immunolocalized in cornicles and in cauda, suggesting a possible new function not related to chemoreception. Moreover, the response of *M. viciae* to different components of the alarm pheromone was assessed by behavioral assays on wingless adult morph; (-)-α-pinene and (+)-limonene were found to be the components mainly eliciting an alarm response. Taken together, our results represent a road map for subsequent in-depth analyses of the OBPs involved in several physiological functions in *M. viciae*, including chemoreception.

## Introduction

Chemical perception in insects is known to be mediated by molecules belonging to the classes of olfactory, gustatory and ionotropic receptors and to the classes of soluble olfactory proteins, odorant-binding proteins (OBPs) and chemosensory proteins (CSPs); however, what these proteins do and how they interact is still not completely clear (Fan et al., 2010; Leal, 2013; Pelosi et al., 2017). In particular, OBPs have long been thought to act exclusively as carriers of chemicals that, once solubilized, were transported to the olfactory receptors (Pelosi et al., 2006; Brito et al., 2016). The generally hydrophobic odorants need to reach the specific receptors bound to the plasma membrane of sensory neuron dendrites, overcoming the hydrophilic barrier that is the sensillar lymph (Pelosi, 1996; Jeong et al., 2013). Several studies performed *in vivo* indicate that OBPs play a key role in chemoreception. RNAi was used to reduce the expression of OBPs in *Anopheles gambiae* and *Culex quinquefasciatus* (Biessmann et al., 2010; Pelletier et al., 2010), in *Drosophila melanogaster* (Swarup et al., 2011) and in *Acyrthosiphon pisum* (Zhang et al., 2017). Results demonstrated that OBPs play a specific role in olfactory perception, suggesting there is a direct correlation between the expression level of OBPs and the ability of insects to perceive odors. Previous studies found that *Drosophila* OBP76a (LUSH) mutants, played an essential role in binding and mediating the recognition of the sex pheromone 11-*cis*-vaccenyl acetate (c-VA) (Xu et al., 2005; Ha and Smith, 2006; Laughlin et al., 2008). These preliminary results should be partially reconsidered in light of more recent research demonstrating that, at sufficiently high concentrations, c-VA is able to activate neuronal stimuli without LUSH (Gomez-Diaz et al., 2013; Li et al., 2014). However, LUSH is still considered a protein that can increase the sensitivity of the c-VA receptor, also protecting pheromone molecules from degradation by ODEs (Gomez-Diaz et al., 2013). Moreover, the capability of LUSH to bind c-VA has been further demonstrated by *in vitro* experiments (Kruse et al., 2003).

It has been demonstrated that deleting OBP28a in *Drosophila melanogaster* basiconic sensilla did not reduce the insect’s ability to respond to olfactory stimuli (Larter et al., 2016), suggesting that OBP28a is not required for odorant transport. Larter and colleagues hypothesize a novel role for OBP, namely, that it modulates odor perception by mitigating the effect of rapid changes in the level of environmental odors. In their model, odorants are transported from the sensillum pore to the sensory neuron through hydrophobic tunnels called pore tubules (Steinbrecht, 1997). However, since in *Drosophila melanogaster* only basiconic sensilla contain pore tubules (Shanbhag et al., 2000), the authors do not exclude that OBP28a expressed in other sensilla type may play different roles including the classical function of odorants carrier (Larter et al., 2016).

Alternatively, different studies suggest that a sensible reduction in olfactory function is related to the reduced levels of certain OBPs (Xu et al., 2005; Biessmann et al., 2010; Pelletier et al., 2010; Swarup et al., 2011). Within the processes relying on chemoreception, it has been proposed that OBPs also play a role in removing chemicals, both those bound to the ORs and those located in the sensory lymph, in order to speed up nervous stimulus termination (Vogt and Riddiford, 1981; Ziegelberger, 1995). That the role of OBPs is related to their binding task is apparent from their multifunctional features, which are not confined to chemical perception (Smartt and Erickson, 2009; Sun Y.L. et al., 2012; Ishida et al., 2013; Pelosi et al., 2017). Indeed, OBPs are expressed in organs that are not connected to chemoreception. In some cases, the same OBP is expressed in chemoreceptive and non-chemoreceptive tissues, suggesting that one type may have multiple roles (Calvello et al., 2003; Li et al., 2008; Sirot et al., 2008; Vogel et al., 2010; Dani et al., 2011; Iovinella et al., 2011; Sun Y.L. et al., 2012; De Biasio et al., 2015). For example, since the same OBPs are expressed in antennae and reproductive organs (Sun Y.L. et al., 2012; Ban et al., 2013), or in antennae and in pheromone glands (Jacquin-Joly et al., 2001; Strandh et al., 2009; Gu et al., 2013; Zhang et al., 2013, 2015; Xia et al., 2015), they may both mediate the recognition of and assisting with the release of the same chemical message. In both cases, the role of OBPs is to solubilize hydrophobic pheromones, binding them in a hydrophilic environment where OBPs are present in high concentration (Nagnan-Le Meillour et al., 2000; Jacquin-Joly et al., 2001; Pelosi et al., 2017).

The different functions imputed to OBPs are in any case linked to the ability of these proteins to bind small hydrophobic molecules, signals of different types originating from different sources. However, the expression of soluble olfactory proteins in chemosensory structures (mainly antennae and mouthparts) indicates that they play a role in chemoreception (Pelosi et al., 2017).

Chemoreception is just one of the roles that OBPs play in aphids (Hemiptera: Aphididae), a group of insects that includes major crop pest in the world. Aphids cause damage directly and indirectly, by feeding and transmitting plant viruses (Nault, 1997; Hogenhout et al., 2008; Webster, 2012). Aphids use their olfactory system and semiochemicals, such as plant volatiles and pheromones, for many purposes: to locate their host plants, select a partner, and escape from danger (van Emden and Harrington, 2007). In aphids, as in other insects, OBPs have the capability to transport semiochemicals across the sensillar lymph toward the ORs located on the sensory neuron membrane (Qiao et al., 2009; Vandermoten et al., 2011; Sun Y.F. et al., 2012). Even if the mode of action of OBPs is not completely understood, the chemical message is known to be transduced into a neuronal impulse that starts at the dendrite of the olfactory sensory neuron (Leal, 2013); next, the signal reaches the antennal lobe in the brain, where it is processed and leads to a behavioral response (Distler and Boeckh, 1996; Fan et al., 2010).

In the present work, we adopted a multidisciplinary approach to study chemoreception in the vetch aphid *Megoura viciae* (Buckton), which feeds exclusively on members of Leguminosae (Nuessly et al., 2004).

After constructing and analyzing the *M. viciae* antennal transcriptome, we identified the OBPs expressed in antennae and determined their expression using the reads per kilobase per million mapped reads (RPKM) method. The expression profile of all the identified OBPs at different developmental stages and in different body parts was also analyzed by RT-qPCR. Moreover, whole mount immunolocalization of five identified OBPs was performed using available antibodies. In addition, scanning electron microscopy (SEM) was carried out on antennae, legs, mouthparts and cauda of both wingless and winged adult morphs to scrutinize the morphology of sensilla expressing the analyzed OBPs at the ultrastructural level. Furthermore, we performed a behavioral assay using the different components of *M. viciae’s* alarm pheromone.

Although our study focuses on the typical chemoreceptive organ, the antennae, and investigates how the expression of OBPs supports the putative role in olfactory and gustatory perception, our results suggest that these soluble proteins play other roles in addition to chemoreception.

## Materials and Methods

### Insect Rearing and Sample Collection

*Megoura viciae* was reared on potted broad bean plants (*Vicia faba* L.) at 24 ± 1°C, 75% ± 5% RH and 16 h light – 8 h dark photoperiod. Aphid cultures were started with insects originally collected from broad bean plants in southern Italy near Salerno (40° 37′ N; 15° 3′ E). In order to synchronize aphid nymphal instars, parthenogenetic females were placed on potted broad bean plants; newborn aphids were separated as soon as they appeared, and adults were removed from plants. Newborn aphids were maintained on plants for 6 days and collected at different developmental stages, from first nymphal instar to adults, both wingless (apterous) and winged (alatae) morphs. Samples were frozen using liquid nitrogen and stored at -80°C until the RNA extraction used for RT-qPCR experiments. Antennae, de-antennaed heads, legs, cornicles, cauda and remaining body parts of wingless adult aphids were dissected under the microscope, fixed and prepared for SEM, immunolocalization experiments, or homogenized in TRI Reagent (Sigma, St. Louis, MO, United States) and stored at -80°C until the RNA extraction used for RT-qPCR experiments. Wingless adults were used in behavior experiments. Some specimens deriving from the described original strain were sent to the Department of Biological Chemistry and Crop Protection, Rothamsted Research, Harpenden, United Kingdom, where aphids were reared in the same conditions described above (24 ± 1°C, 75% ± 5% RH and 16 h light – 8 h dark photoperiod). Antennae cut from wingless adults were used for RNA extraction and sequencing at the Beijing Genomics Institute (BGI).

### Scanning Electron Microscopy (SEM)

Adult aphids (6 in the wingless morph and 2 in the winged morph) were prepared as described by Sun et al. (2013). Briefly, they were fixed in 70% ethanol for 2 h and cleaned in an ultrasonic bath for 1 min in the same solution. Finally, samples were dehydrated in 100% ethanol for 30 min, air-dried, coated in gold by K250 sputter coater (Emitech, Ashford, Kent, United Kingdom) and examined with SEM-FEG XL-30 microscope (Philips, Eindhoven, The Netherlands).

### Total RNA Extraction and cDNA Synthesis

Total RNA, collected from 800 antennae, 80 de-antennaed heads, 500 legs, 500 cornicles-cauda and 40 remaining body parts of wingless adult aphids and from 30 aphids of each different nymphal instar (I, II, III, IV) and each adult morph, was extracted using TRI Reagent (Sigma, St. Louis, MO, United States), following the manufacturer’s instructions. The concentration of total RNA was measured spectrophotometrically at 260 nm, using a NanoDrop ND-1000 instrument (Nanodrop Technologies, Wilmington, DE, United States). The purity of RNA was estimated at absorbance ratios OD260/280 and OD260/230, and the integrity was verified on 0.8% agarose gel electrophoresis. In order to efficiently remove genomic DNA contamination, the samples were treated with 1U of DNase I (Deoxyribonuclease I, Amplification Grade, Invitrogen-Life Technologies, Carlsbad, CA, United States) per microgram of RNA for 15 min at room temperature, following the manufacturer’s guidelines. cDNA was synthesized using the SuperScript III First-Strand Synthesis System for RT-qPCR (Invitrogen-Life Technologies), according to the manufacturer’s protocol, using 5 μg of total RNA per sample. The cDNA synthesis reaction was diluted with nuclease-free water to a final volume of 100 μl and immediately used for RT-qPCR studies or stored at -20°C.

### RNA-Seq Data Generation and *de novo* Transcriptome Assembly

Antennal transcriptome sequencing was performed with poly(A)enriched mRNA fragmented to an average of 150 nucleotides. Sequencing was carried out by the BGI using paired-end sequencing on an Illumina HiSeq2000 sequencer.

After transformation to raw data, low quality (reads with unknown sequences ‘N’) adaptor sequences were removed; reads with certain lengths of overlap were combined to form longer fragments, called contigs. These contigs were subjected to further processing of sequence clustering to form longer sequences without N. Such sequences were defined as unigenes.

Reads were trimmed of adapters using Cutadapt (Martin, 2011), and of bad quality regions using Sickle (Joshi and Fass, 2011). Subsequently, reads were assembled using Trinity 2.2 with default parameters (Grabherr et al., 2011).

### Annotation of OBP Coding Transcripts

The base of the annotation was a hand-curated database of OBP proteins which, among others, contained known aphid candidate protein sequences. The assembled sequences were compared with the references dataset using blastx. All sequences that generated a hit were further scrutinized by blastx comparison against the NCBI non-redundant database (nr), removing any sequences with evidence for an identity that differs from OBP. Finally, the remaining candidates were translated and aligned with the references using MAFFT (Katoh and Standley, 2013), removing candidates that did not align well with known OBP protein sequences. During this step, candidates were also scrutinized for the presence of the conserved OBP cysteine-pattern.

### Quantitative Real Time PCR (RT-qPCR)

RT-qPCR experiments were carried out in a 7500 Fast Real-Time PCR System (Applied Biosystems- Life Technologies, Carlsbad, CA, United States), on cDNA samples prepared from 5 different nymphal instars, including winged and wingless morphs, and from different body parts (antennae, de-antennaed heads, legs, cornicles and cauda and remaining body parts) of wingless adults. Ribosomal protein S9 (*RPS9*) and ribosomal protein L32 (*RPL32*), whose use was validated in a previous work (Cristiano et al., 2016), were chosen as reference genes for the normalization of data obtained from aphids of different nymphal instars and aphids’ different body parts RT-qPCR, following the guidelines reported in minimum information required for publication of quantitative real-time PCR experiments (MIQE) (Bustin et al., 2009) and minimum information necessary for quantitative real-time PCR experiments (Johnson et al., 2014). Specific primers were designed for each *M. viciae* OBP gene and for the reference genes, using Primer Express v3.0 software (ABI, Foster City, CA, United States). Primers of about 20 bp, with approximately 50% G/C content, were selected (**Table 1**). PCR amplifications were performed using GoTaq qPCR Master Mix (Promega, Madison, WI, United States). The reactions were carried out in a 20 μl final volume containing 5 μl of diluted first-strand cDNA (60 ng/μl) and 0.3 μmol/L primer final concentration. Cycling conditions for all genes were: 2 min at 95°C, 40 cycles of 15 s at 95°C and 1 min at 60°C. At the end of each run, a melting curve analysis was performed in order to confirm the specificity of PCR products. All amplification reactions were run in triplicate (technical replicates) and included negative controls (no template reactions, replacing cDNA with H_2_O). All the experiments were performed for a set of 3 biological replicates. In order to evaluate gene expression levels, relative quantification was performed using the equations described by Liu and Saint (2002), based on PCR amplification efficiencies of reference and target genes. Amplification efficiency of each target gene and of *RPS9* and *RPL32* was determined according to the equation *E* = 10^-1/S^ -1 (Lee et al., 2006), where S is the slope of the standard curve generated from 4 serial 10-fold dilutions of cDNA. All data (mean ± SD) were compared by one-way analysis of variance (ANOVA) and Tukey’s HSD multiple comparisons test using GraphPad Prism 6.00 software for Windows (GraphPad Software, La Jolla, CA, United States^1^). Significant differences were expressed in terms of *p*-value (^∗^*p* < 0.05, ^∗∗^*p* < 0.01, ^∗∗∗^*p* < 0.001).

**Table 1:** Primers used for RT-qPCR.

Gene Name	Primer sequence (5′-3′)
*MvicOBP1*	F: ACCACATTGTTAACGACGGC
	R: GTTGCGGCTAACTCACACTC
*MvicOBP2*	F: CCAAGCCAACAATGACCGAA
	R: GCCTTCTTGTGTTCGTCTGG
*MvicOBP3*	F: CTAGGACTGCTGAACGACGA
	R: CAGACATGCCATCACAGTGT
*MvicOBP4*	F: ACGTAGAGTTGCAGGGTGTT
	R: TCGAAACTTTTGGAGGGCTG
*MvicOBP5*	F: AGTAGCAGCTGACGAGTGTT
	R: CGTCTTCGGTGAGCAAATGA
*MvicOBP6*	F: GAAAAGAGCCACCATGTCTT
	R: TTGGGGCAGCTCATATACAT
*MvicOBP7*	F: TTGCGACGCTTACTTGAGTG
	R: TGTTGTTGTTGTCCTCCGGA
*MvicOBP8*	F: TGATGGGTTGCCTGATGAGA
	R: AAGTTGTCACAATTCCGGCC
*MvicOBP9*	F: TGCCGGAGAAGAACTTGGAA
	R: CCTTCAGTGCTGGTGATTCC
*MvicOBP10*	F: AGTGTTGCTTAGACGAGATGT
	R: AACAAAAGCCGCTTCCAAAC
*RPS9*	F: TTCTGGGAGTCCAAACGAAC
	R: TCTTGGAACGCAGACTTCAA
*RPL32*	F: ATGCTGCCTTCCAAATTCCG
	R: ACGTGCATTTCCATTGGTCA

*F, forward primer; R, reverse primer; *RPS9, RPL32*, reference genes.*

### Whole-Mount Immunolocalization Experiments

For this assay, only the wingless morphs, collected at the first day of the adult stage, were considered. In particular, 6 antennae, 6 mouthparts, 6 cornicles and 6 caudae from wingless specimens were dissected under the microscope and washed twice with PBS, pH 7.4. Given that winged aphids are rare and difficult to recover and maintain in breeding they were not considered for this analysis. After the washing step, samples were fixed in 4% paraformaldehyde in PBS for 2 h and then washed twice with the same buffer. Samples were then incubated for 30 min with PBS containing 2% BSA (to reduce non-specific binding) and 0.1% of the detergent Tween 20 (Sigma) to permeabilize tissues favoring the entrance of antibodies. Samples were then incubated for 1 h at room temperature with antisera raised in rabbit, diluted 1:200. Whole mount immunolocalization experiments were carried out on five among the ten identified OBPs because only five antibodies were already available. We used antisera against OBPs 1, 3, 6, 7, and 8 of *A. pisum* since they are ortholog genes of *M. viciae* OBPs (Zhou et al., 2010). Antibodies, kindly provided by Prof. Paolo Pelosi (University of Pisa), were produced against the entire amino acid OBP sequences and they were not affinity purified. Since recombinant OBPs were not available for pre-adsorption controls against OBP antibodies, we validated their specificity by western blot using protein extract from the whole *M. viciae* body (**Supplementary Figure S1**). Briefly, we used 20 μg of proteins (each lane), separated by a 12% polyacrylamide gel electrophoresis and transferred on a Whatman nitrocellulose membrane. Anti-OBP antibodies were diluted 1:1000 in tris-buffered saline and 0.1% Tween 20 (TBS-T) with 5% bovine serum albumin (BSA). Goat anti-rabbit antibodies conjugated to horseradish peroxidase, diluted 1:15000 in TBS-T, was used as a secondary antibody after a pre-absorption using an extra lane loaded with protein extracted from aphid whole body. For detection, enhanced chemo luminescence (ECL) was used and signals were measured with Chemidoc^TM^ MP System.

These antibodies have been previously used in experiments on the pea aphid *A. pisum* OBPs (De Biasio et al., 2015) and on the peach aphid *Myzus persicae* OBPs (Sun et al., 2013), that are orthologs of *A. pisum* OBPs (Zhou et al., 2010). We confirmed the high similarity level among *A. pisum* and *M. viciae* OBPs by amino acid alignment reported in **Supplementary Figure S2**.

Samples were washed with PBS and incubated for 1h in a dark chamber with the secondary goat anti-rabbit tetramethylrhodamine (TRITC)-conjugated antibody diluted 1:200 (Jackson, Immuno Research Laboratories Inc., West Grove, PA, United States) in blocking solution containing 0.1% Tween 20. In all controls, primary polyclonal anti-OBPs antibodies were omitted or substituted with rabbit pre-immune serum (1:200), and sections were treated with blocking solution containing 0.1% Tween 20 (Sigma) and incubated only with the secondary antibody. Coverslips were mounted with City fluor (City fluor Ltd., London, United Kingdom), and immunofluorescence was analyzed using an inverted laser-scanning confocal microscope (TCS SP5, Leica Microsystems, Wetzlar, Germany) equipped with a HCX PL APO lambda blue 63.0 × 1.40 NA OIL UV objective. Images were acquired using the Leica TCS software (emission windows fixed in the 551–626 range) without saturating any pixel. Z-stack sections acquisition was carried out by selecting the optimized acquisition parameters. The displayed bright field and fluorescent images represent Z-stack projections of sections obtained with the open source image software Fiji (average intensity) (Schindelin et al., 2012). Fluorescence and bright field images were combined with Adobe Photoshop (Adobe Systems Incorporated, San Jose, CA, United States).

### Behavioral Assays

The behavioral response of *M. viciae* to the components of the alarm pheromone was investigated under the conditions reported in Sun Y.F. et al. (2012) for *A. pisum*, using a Y-tube. Briefly, an airflow of 0.5 L/min was introduced into each arm of the glass Y-tube olfactometer through a glass stimulus chamber (odor source adapter) attached to each of the two arms. In each test, 1 μl of hexane solution of each chemical compound, concentration 0.5%, was placed in the glass stimulus chamber of the “treatment” arm. As a control, 1 μl of hexane was placed in the glass stimulus chamber of the “control” arm of the olfactometer. Groups of twenty wingless adult aphids were introduced at the bottom of the Y-shaped copper wire and allowed to walk to either arm at the Y-junction. After 15 min, the number of aphids in the treatment and control sides of the olfactometer were counted. Six replications with each compound were performed. Tested compounds were (E)-β-farnesene (Bedoukian Research, Danbury, CT, United States), (±)-α-pinene, β-pinene, (-)-α-pinene, (+)-limonene, hexane (Sigma-Aldrich-Fluka) and a mixture comprising (E)-β-farnesene 14.2%, (-)-α-pinene 11.8% and β-pinene 74% (Francis et al., 2005). The behavioral responses to all the analyzed compounds and mixture were compared by one-way analysis of variance (ANOVA) and Tukey’s HSD multiple comparisons test using GraphPad Prism 6.00 software for Windows (GraphPad Software) (^∗^*p* < 0.05, ^∗∗^*p* < 0.01, ^∗∗∗^ *p* < 0.001).

## Results

### Scanning Electron Microscopy of Sensilla

**FIGURE 1:**
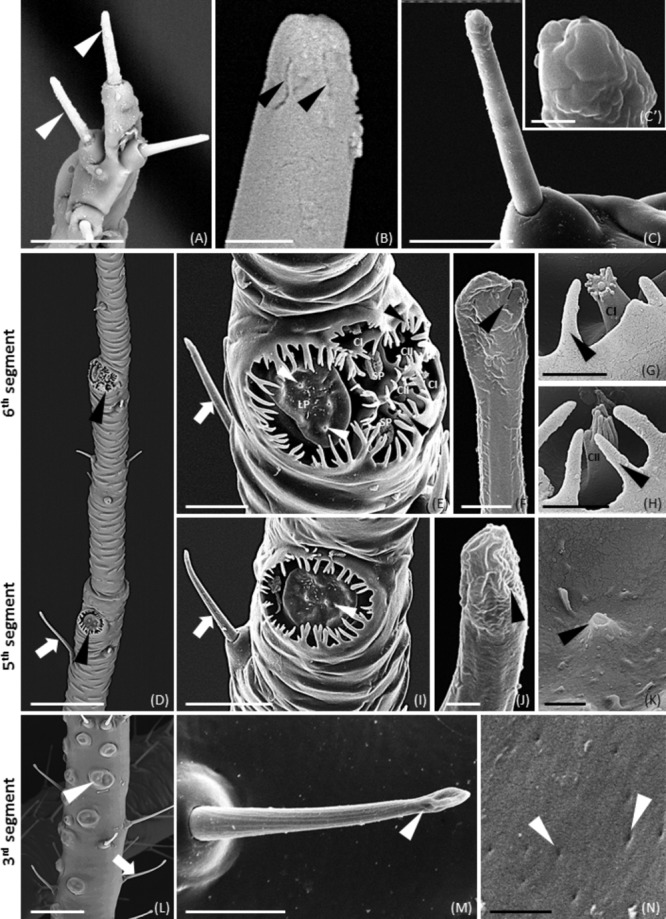
SEM images showing the distribution and morphology of different sensilla on wingless *M. viciae* antennae. **(A–C)** Type II trichoid sensilla located on the terminal part of the antenna (arrowheads in **(A)**), and on processus terminalis **(C)** showing a blunt tip with a grooved surface **(B,C’)**. **(D)** Global view of primary rhinaria on 5 and 6th segments (arrowheads) with a type I trichoid sensilla (arrow). **(E)** Details of the primary rhinaria on the 6th segment composed of 1 large placoid sensillum (LP) with porous structures (white arrowheads), 2 small placoid sensilla (SP), and 4 coeloconic pegs surrounded by cuticular fringes (black arrowheads). **(G,H)** Detail of type I (CI in **(G)**) and type II (CII in **(H)**) coeloconic sensilla in the 6th segment surrounded by cuticular fringes (arrowheads). **(I)** Detail of placoid sensillum of 5th segment. Porous structures were visible on the flat surface (arrowhead in **(K)**). **(L)** Placoid sensilla forming the secondary rhinaria of the 3rd segment (arrowhead) and trichoid sensilla (arrow). **(F,J,M)** Details of type I trichoid sensilla showing a groove surface and porous structures on the tip. **(N)** Detail of a placoid sensillum with a smooth surface not surrounded by cuticular fringes and small pores on the flat surface. Bars in **(A,E,I,M)**, 10 μm; bars in **(B,F–H,K)**, 1 μm; bar in **(C)**, 5 μm; bars in **(C’,J,N)**, 500 nm; bars in **(D,L)**, 50 μm.

**FIGURE 2:**
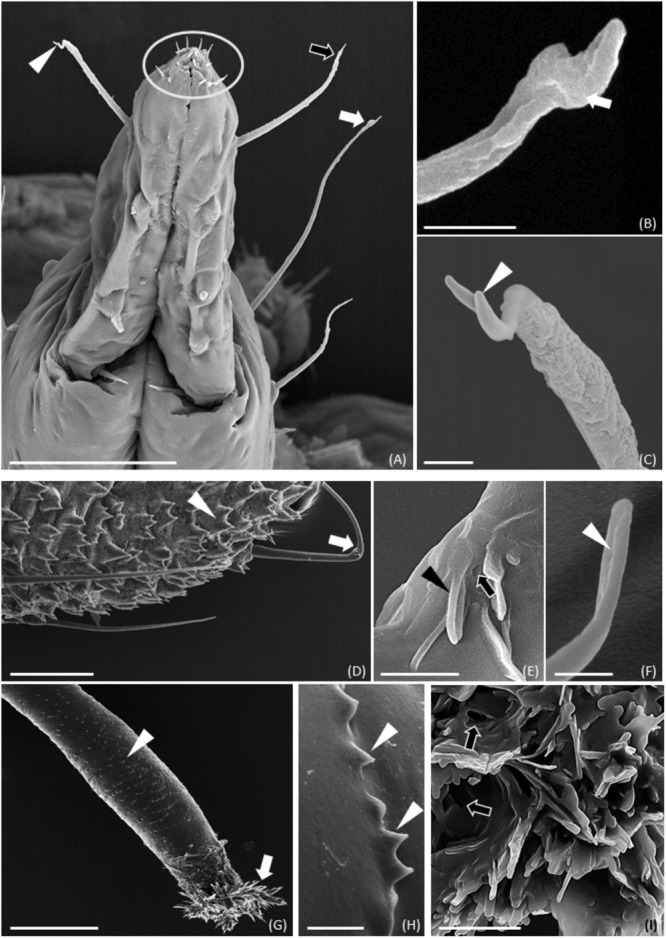
SEM images showing sensilla on *M. viciae* mouthparts, cauda and cornicles. **(A)** Long sensilla symmetrically distributed (arrowhead) and short sensilla (encircled) situated on the labium tip. **(B)** Detail of long sensilla tip with pre-apical expansion (black arrow in **(A)**) or in the shape of a cup (arrow in **(A,B)**). **(C)** Detail of long sensilla branched tip (arrowhead). **(D–F)** Detail of porous (arrow in **(E)**) or fissure like structures (arrowheads in **(E,F)**) on long sensilla and finger-like extensions (arrowhead in **(D)**) on *M. viciae* cauda. Finally, SEM observation highlights the presence of cuticular finger-like structures (arrowheads in **(G,H)**) on cornicle surface. Moreover, hole-like structures are evident among cuticular tufts (arrows in **(I)**) of cornicle terminal region. Bar in **(A)**, 50 μm; bars in **(B,E,F)**, 1 μm; bar in **(C)**, 2 μm; bar in **(D)**, 25 μm; bar in **(G)**, 100 μm; bars in **(H,I)**, 5 μm.

Scanning electron microscopy observations of *M. viciae* highlighted differences of legs and antennae both in the morphology and in the distribution of sensilla (**Supplementary Figures S3A–D** and **Figures 1A–N**). In legs, numerous trichoid sensilla, uniform in size, shape and distribution, were visible. In the vetch aphid, sensilla showed a peak with a rounded shape, without pores (**Supplementary Figures S3A,B,D**). SEM images show the insertion of the sensillum basal portion in a cuticular extension on the leg (**Supplementary Figure S3C**). On the antennae of both wingless (**Figures 1A–N**) and winged morph (**Supplementary Figures S4A–H**), different types of sensilla were recognizable, depending on the segment. Type II trichoid sensilla were located on the antenna tip of the 6th segment and along the processus terminalis on the same segment. Type II trichoid sensilla located on the antenna tip appeared as short hairs with a blunt tip showing fissure-like structures and grooves (**Figures 1A,B** and **Supplementary Figures S4A,A’**). Type II trichoid sensilla on the processus terminalis (**Figures 1C,C**’ and **Supplementary Figures S4B,B’**), and type I trichoid sensilla, visible from the base of the antenna to the 6th segment, were characterized by a grooved surface and a swollen tip with fissure-like and porous structures (**Figures 1D–F,I,J,L,M** and **Supplementary Figures S4C,F,F’,G,H**). Primary rhinaria were clearly observable on the 5th and 6th antennal segments (**Figure 1D** and **Supplementary Figures S4C,F**). In particular, a placoid sensillum was located in the distal end of the 5th segment (**Figures 1D,I** and **Supplementary Figure S4F**), while on the 6th segment 1 large placoid sensillum, 2 smaller ones, 2 type I and 2 type II coeloconic sensilla were distinguishable and surrounded by cuticular fringes (**Figures 1D,E,G,H** and **Supplementary Figures S4C–E**). The placoid sensilla appeared as circular plates showing porous structures on their flat surface (**Figures 1E,I,K** and **Supplementary Figures S4E,F**). On the 3rd antennal segment, secondary rhinaria were constituted by about 30 placoid sensilla in the wingless aphids (**Figure 1L**) and of about 60 placoid sensilla in the winged morph (**Supplementary Figures S4G,H**), both showing a smooth ridge not surrounded by cuticular fringes and small pores on their flat surface (**Figure 1N**). Moreover, we found that in the winged aphids the 3rd segment was longer than in wingless morph (1040 μm instead of 743 μm). Both the wingless and winged vetch aphid presented sensilla associated with mouthparts and caudal region. Since no differences between the two morphs were found, only data of winged morph were shown (**Figures 2A–I**). In the mouthparts, these sensilla showed different morphologies: they had pre-lobed apical extensions (**Figure 2B**) or branched tips (**Figure 2C**). Numerous short sensilla, arranged symmetrically, were evident on the labium end part (**Figure 2A**). SEM observations of the cauda (**Figures 2D–F**) showed the presence of long sensory hair-like structures with small pores (**Figure 2E**) or a fissure-like structure (**Figure 2F**). The entire caudal surface was covered by numerous finger-like projections arranged in groups (**Figure 2D**). Similar structures were also found on the surface of cornicles (**Figures 2G,H**). In addition, the terminal region of cornicles was characterized by the presence of cuticular fingers among which holes were visible (**Figure 2I**).

Scanning electron microscopy observations of *M. viciae* legs and antennae highlighted differences both in the morphology and in the distribution of sensilla. In legs, numerous trichoid sensilla were visible. On the antennae of both wingless and winged morph type II trichoid sensilla, type I trichoid sensilla, primary rhinaria (5th and 6th segments) and secondary rhinaria (3rd segment) were found. Moreover, the vetch aphid presented sensilla associated with mouthparts and caudal region.

### Identification of OBP Candidates

First, putative OBP coding sequences needed to be identified. To this end, RNA sequencing of *M. viciae* antennae was performed. Sequencing data were assembled using the Trinity assembler, resulting in 43,251 predicted transcripts from 36,239 ‘genes’. The N50 of the assembled transcripts was 2,063 bp, with a corresponding median contig length of 571 bp, average of 1,115 bp and 48,243,578 total nucleotides in the assembly. The assembled data were used in the identification and annotation of ten candidate OBP genes, named *MvicOBP1, MvicOBP2, MvicOBP3, MvicOBP4, MvicOBP5, MvicOBP6, MvicOBP7, MvicOBP8, MvicOBP9*, and *MvicOBP10*. The nucleotide sequences were deposited in GenBank under the accession numbers listed in **Table 2**. OBPs expression level in antennae was estimated as reads per kilobase per million mapped reads (RPKM).

**Table 2:** Candidate OBP genes in *Megoura viciae* antennae.

Unigene reference	Gene name	ORF (bp)	Accession number	BLASTx annotation	*E*-value	AA Identity (%)	Antennae RPKM value
4148_c0_g2_i1	*MvicOBP1*	480	MG596881	[NP_001153526.1] Odorant-binding protein 1 precursor [*Acyrthosiphon pisum*]	2e-111	99	3.81028
3537_c0_g1_i1	*MvicOBP2*	726	MH177887	[NP_001153528.1] Odorant-binding protein 2 precursor [*Acyrthosiphon pisum*]	5e-165	95	7.56772
20255_c0_g1_i1	*MvicOBP3*	426	MG596882	[NP_001153529.1] Odorant-binding protein 3 precursor [*Acyrthosiphon pisum*]	4e -92	96	4.57565
10025_c0_g1_i1	*MvicOBP4*	600	MH177888	[NP_001153530.1] Odorant-binding protein 4 precursor [*Acyrthosiphon pisum*]	7e-128	93	5.65031
5845_c0_g1_i1	*MvicOBP5*	666	MH177889	[NP_001153531.1] Odorant-binding protein 5 precursor [*Acyrthosiphon pisum*]	3e-152	95	6.1596
9875_c1_g1_i3	*MvicOBP6*	648	MG596883	[NP_001153532.1] Odorant-binding protein 6 [*Acyrthosiphon pisum*]	8e-104	95	2.75666
5098_c0_g1_i1	*MvicOBP7*	468	MG596884	[NP_001153533.1] Odorant-binding protein 7 precursor [*Acyrthosiphon pisum*]	4e-96	88	3.92025
18200_c0_g1_i1	*MvicOBP8*	486	MG596885	[NP_001153534.1] Odorant-binding protein 8 precursor [*Acyrthosiphon pisum*]	2e-96	95	4.3177
594_c0_g1_i1	*MvicOBP9*	501	MH177890	[NP_001153535.1] Odorant-binding protein 9 precursor [*Acyrthosiphon pisum*]	7e-102	90	6.26894
23913_c0_g1_i1	*MvicOBP10*	435	MH177891	[NP_001153525.1] Odorant-binding protein 10 [*Acyrthosiphon pisum*]	8e-58	81	5.65906

Among the ten identified candidate OBP genes, *MvicOBP1, MvicOBP3, MvicOBP6, MvicOBP7* and *MvicOBP8* were selected for immunolocalization analysis because antibodies were already available. Antibodies against *A. pisum* OBPs were used because of the high sequence similarity among the selected *M. viciae* OBPs and the same *A. pisum* OBPs (**Supplementary Figure S2**). The alignment of the 10 identified antennal *M. viciae* OBPs is shown in **Supplementary Figure S5**.

RNA sequencing and assembly of *M. viciae* antennae allowed the identification and the annotation of ten candidate OBP genes. *MvicOBP1, MvicOBP3, MvicOBP6, MvicOBP7*, and *MvicOBP8* were selected for immunolocalization analysis because specific antibodies were already available.

### OBP Expression Patterns in Different Body Parts and Nymphal Instars of *M. viciae*

**FIGURE 3:**
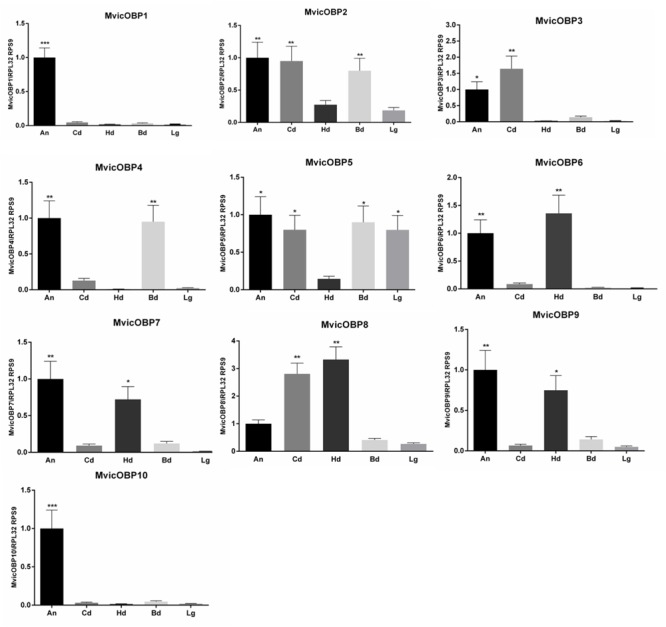
Relative expression level of *M. viciae* OBPs in different body parts. OBP expression levels were quantified by RT-qPCR. Bars represent the standard deviation of the mean for 3 independent experiments. Significant differences are denoted by asterisks (Tukey’s test, (^∗^*p* < 0.05, ^∗∗^*p* < 0.01, ^∗∗∗^*p* < 0.001)). Lg, legs; Cd, cornicles-cauda; Hd, head; Bd, body; An, antennae. Reference genes: *RPL32, RPS9*. Calibrator sample: antennae.

In order to evaluate the expression level in different body parts of all the ten identified *M. viciae* OBPs, RT-qPCR experiments were carried out using gene-specific primers and using *RPS9* and *RPL32* as reference genes. We validated the use of these reference genes in RT-qPCR experiments on different developmental stages of *M. viciae*, in a previous work (Cristiano et al., 2016) and we repeated the validation step on the analyzed different body parts observing that the expression levels of *RPS9* and *RPL32* remained the same (**Supplementary Figure S6**). **Supplementary Figure S7** shows the OBPs relative expression calibrated on *RPS9* and *RPL32*, respectively. RT-qPCR results showed that *MvicOBP1* and *MvicOBP10* transcripts were significantly more expressed in *M. viciae* antennae than in the other body parts (^∗∗∗^*p* < 0.001). Transcripts coding for *MvicOBP2* were more expressed in antennae, cauda and bodies than in heads and legs (^∗∗^*p* < 0.01), while transcripts for *MvicOBP3* were significantly more expressed in antennae (^∗^*p* < 0.05) and in cauda (^∗∗^*p* < 0.01). For *MvicOBP4* the statistically highest transcript levels were observed in antennae and bodies (^∗∗^*p* < 0.01), while the expression levels of *MvicOBP5* were statistically the same in antennae, cauda, bodies and legs (^∗^*p* < 0.05). For *MvicOBP6* and *MvicOBP7*, the statistically highest transcript expression levels were observed in antennae (^∗∗^*p* < 0.01) and in heads (^∗∗^*p* < 0.01 for *MvicOBP6* and ^∗^*p* < 0.05 for *MvicOBP7*). Moreover, we found that the gene encoding for *MvicOBP8* was statistically mainly expressed in the cauda and in heads (^∗∗^*p* < 0.01), while *MvicOBP9* transcripts were more expressed in antennae (^∗∗^*p* < 0.01) and heads (^∗^*p* < 0.05) (**Figure 3**).

**FIGURE 4:**
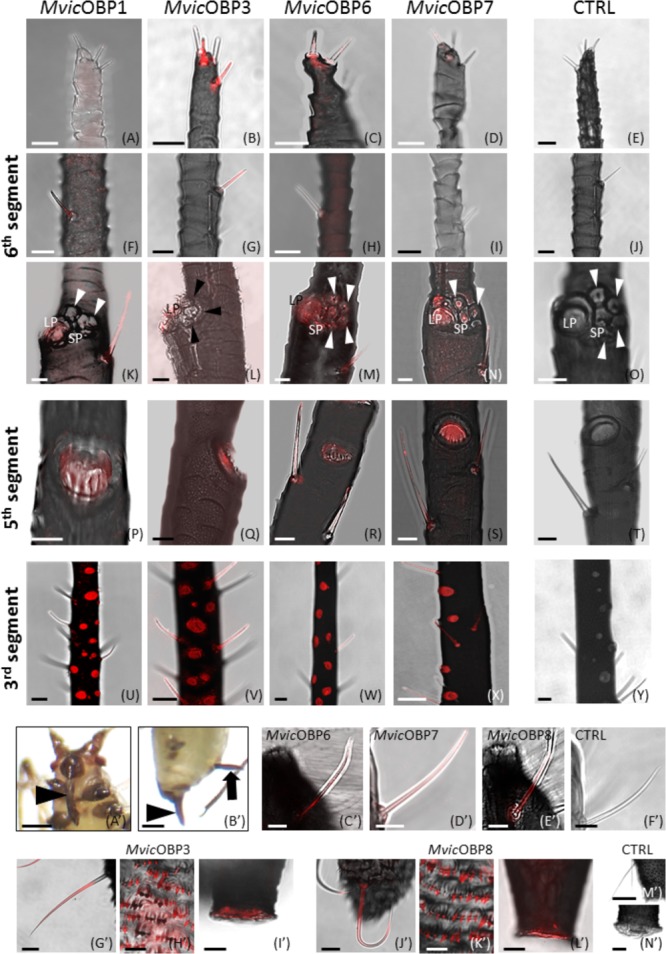
**(A–Y)** Whole-mount immunolocalization experiments showing the OBP expression in type II trichoid sensilla located on the antennal tip **(A–D)**, in type II trichoid sensilla on the 6th antennal segment **(F–I)**, in primary rhinaria on the 5th **(K–N)** and 6th segments **(P–S)** and in secondary placoid sensilla on the 3rd segment **(U–X)**. **(E,J,O,T,Y)** Negative controls. Bars in **(A–T)**, 10 μm; bars in **(U–Y)**, 25 μm. **(A’–N’)** Whole-mount immunolocalization experiments showing the OBP localization in the mouthparts (arrowhead in **(A’)**), in the cauda (arrowhead in **(B’)**) and in cornicles (arrow in **(B’)**).**(E–G,C’–E’)** Immunolocalization of OBPs in the long sensilla on the labium sides. **(G’–L’)** OBPs detection in hair-like structures and finger-like projections in cauda and in cornicles. **(F’,M’,N’)** Negative controls. Bars in **(A’,B’)**, 250 μm; bars in **(C’–F’,H’,K’)**, 10 μm; bars in **(M’)**, 50 μm; bars in **(G’,I’,J’,L’,N’)**, 20 μm

RT-qPCR experiments were confirmed by whole-mount immunolocalization experiments carried out on five OBPs for which antibodies were available (**Figure 4**). In particular, *MvicOBP1, MvicOBP3, MvicOBP6*, and *MvicOBP7* were immunolocalized in type II trichoid sensilla (**Figures 4A–I**) and in the primary rhinaria located on the 5th and 6th segments of antenna (**Figures 4K–S**). *MvicOBP1* was expressed mainly in the lymph of type I trichoid sensilla located on the 6th segment (**Figures 4F–K**). Moreover, *MvicOBP1* was expressed on placoid sensilla located on the 3rd, 5th, and 6th antennal segments (**Figures 4K,P,U**). *MvicOBP3* was expressed in the lymph of type II trichoid sensilla located on the distal region of the antenna (**Figures 4B,G**) and in the large placoid sensilla on the 6th segment (**Figures 4L**). Moreover, *MvicOBP3* was expressed in placoid sensilla on the 5th and 3rd segments (**Figures 4Q,V**). In contrast, the small placoid sensilla and the coeloconic sensilla on the 6th segment were not labeled by the antiserum against *MvicOBP3* (**Figure 4L**). *MvicOBP6* was immunolocalized in the lymph of all sensilla located on the 3rd, 5th, and 6th antennal segments, except in type I trichoid sensilla, and in the 6th segment coeloconic sensilla (**Figures 4C,H,M,R,W**). Finally, placoid and trichoid sensilla on the 3rd and 5th segments and the lymph of type II trichoid sensilla, placoid and coeloconic sensilla on the 6th segment were labeled specifically by the antibody against *MvicOBP7*, while type I trichoid sensilla on the 6th segment were not stained by this antibody (**Figures 4D,I,N,S,X**). In none of sensilla described above, we found the expression of *MvicOBP8* (**Supplementary Figure S8A**). The expression profile of OBPs in the mouthparts (**Figure 4A’**) and in the terminal body part (**Figure 4B**’) is shown in **Figures 4C’–E’,G’–L’**. In the mouthparts, *MvicOBP6, MvicOBP7* and *MvicOBP8* were expressed in the inner lymph of hair-like sensilla (**Figures 4C’–E’**). In contrast, no signal was detected for *MvicOBP1* and *MvicOBP3* (**Supplementary Figures S8C,F**). *MvicOBP3* and *MvicOBP8* were detected in the hair-and finger-like structures of the terminal region of the body and in the cornicles (**Figures 4G’–L’**), while in both these regions no signals were found for *MvicOBP1, MvicOBP6, MvicOBP7* (**Supplementary Figures S8D,E,G–J**). No signal was detected in control experiments in which the primary antibodies were substituted with the rabbit pre-immune serum (**Figures 4E,J,O,T,Y,F’,M’,N’**) or omitted (**Supplementary Figure S8B**).

**Table 3** summarizes the localization of the five analyzed *MvicOBPs* in different sensilla types in the wingless morph.

**Table 3:** Immunolocalization of five among the ten identified *MvicOBPs* in different body parts.

	Immunolocalization			
	Antennae	Mouthparts	Cauda	Cornicles
*Mvic*OBP1	- Type II trichoid sensilla, 5 and 6th segments - Type I trichoid sensilla, 6th segment - Primary rhinaria - Secondary rhinaria	None	None	None
*Mvic*OBP3	- Type II trichoid sensilla, antennal tip - Type II trichoid sensilla, 5 and 6th segments - Primary rhinaria - Secondary rhinaria	None	Hair- and finger-like structures of the terminal region	Detected
*Mvic*OBP6	- Type II trichoid sensilla, antennal tip - Type II trichoid sensilla, 5 and 6th segments - Primary rhinaria - Secondary rhinaria	Hair-like sensilla	None	None
*Mvic*OBP7	- Type II trichoid sensilla, 5 and 6th segments - Primary rhinaria - Secondary rhinaria	Hair-like sensilla	None	None
*Mvic*OBP8	None	Hair-like sensilla	Hair- and finger-like structures of the terminal region	Detected

**FIGURE 5:**
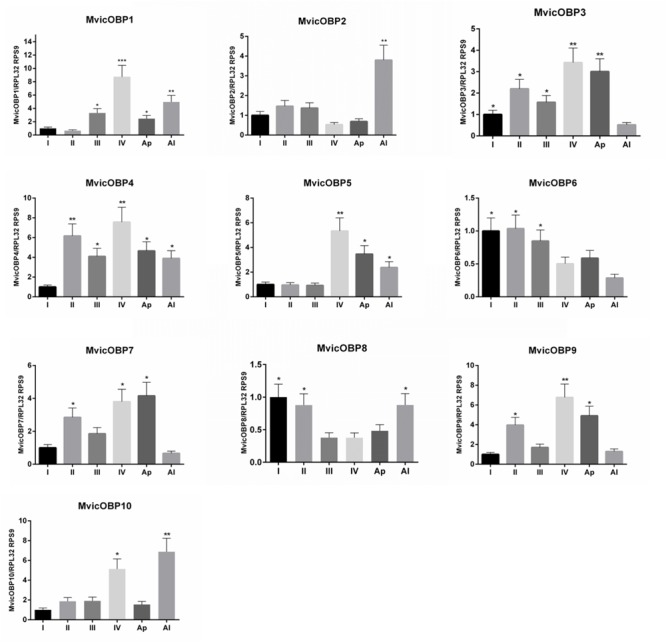
Relative expression level of *M. viciae* OBPs in different nymphal instars. OBP expression levels were quantified by RT-qPCR. Bars represent the standard deviation of the mean for 3 independent experiments. Significant differences are denoted by asterisks (Tukey’s test, (^∗^*p* < 0.05, ^∗∗^*p* < 0.01, ^∗∗∗^*p* < 0.001)). I, 1st nymphal instar; II, 2nd nymphal instar; III, 3rd nymphal instar; IV, 4th nymphal instar; Ap, winged adults; Al, winged adults. Reference genes: *RPL32, RPS9*. Calibrator sample: 1st nymphal instar

RT-qPCR was also used to investigate on the OBPs expression levels in different nymphal instars. Results showed that *MvicOBP1* transcripts were significantly more expressed in the IV nymphal instar (^∗∗∗^*p* < 0.001), in the winged adults (^∗∗^*p* < 0.01) and both in the wingless adults and III nymphal instar (^∗^*p* < 0.05). *MvicOBP2* transcripts were significantly more expressed in the winged morph (^∗∗^*p* < 0.01). Transcripts encoding for *MvicOBP3* showed high expression levels in the IV nymphal instar and in the wingless adults (^∗∗^*p* < 0.01), which agrees with the lower levels of expression observed in the early nymphal instars (^∗^*p* < 0.05) and in the winged adults. *MvicOBP4* transcripts were more expressed in the II and IV nymphal instar (^∗∗^*p* < 0.01), while expression of *MvicOBP5* was statistically higher only in the IV nymphal instar (^∗∗^*p* < 0.01). *MvicOBP6* transcripts were found to be more expressed in the early nymphal instars (I, II, III) (^∗^*p* > 0.05). Equally, the levels of transcription of the gene encoding for *MvicOBP8* were statistically higher in the first two pre-productive stages (I and II) and in the winged adult morph (^∗^*p* > 0.05). The expression of the gene encoding for *MvicOBP7* was higher both in the II and IV nymphal instar and in the wingless adult stage (^∗^*p* > 0.05), but lower in the other immature stages (I, III) and in winged. Equally, transcripts encoding for *MvicOBP9* were more expressed in the IV instar (^∗∗^*p* < 0.01) and in the II and wingless morph (^∗^*p* < 0.05). The expression of the gene encoding for *MvicOBP10* was higher both in the IV nymphal instar (^∗^*p* < 0.05) and in winged adult (^∗∗^*p* < 0.01) (**Figure 5**).

All the ten identified *MvicOBPs* were analyzed by RT-qPCR in different body parts and in all the developmental stages. *MvicOBP1, MvicOBP3, MvicOBP6, MvicOBP7* and *MvicOBP8* were selected for further analysis of immunolocalization showing a complex immunolocalization pattern in all the analyzed body parts (antennae, mouthparts, cornicles and cauda).

### Behavioral Experiments

**FIGURE 6:**
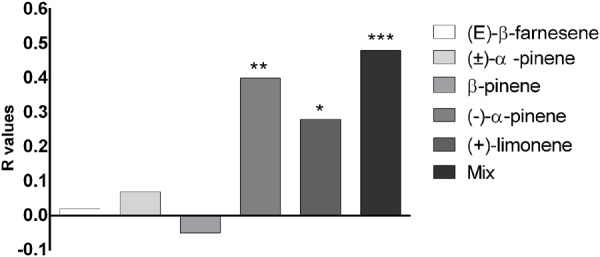
Behavioral responses of *M. viciae* to the main compounds identified in the insect’s cornicle secretions and to the mixture containing (E)-β-farnesene 14.2%, (–)-α-pinene 11.8%, β-pinene 74%. The repellency index R was calculated by the formula *R* = (C–T)/(C+T), where T indicates the number of aphids in the arm with the compound to be tested and C, those in the control arm. Asterisks indicate that the repellence observed is significantly different from the control (^∗^*p* < 0.05, ^∗∗^*p* < 0.01, ^∗∗∗^*p* < 0.001, Student’s *t*-test)

Behavioral experiments on *M. viciae* wingless adults were performed with the main compounds identified in a cornicle droplet ((E)-β-farnesene, β-pinene, (-)-α -pinene and (+)-limonene). For the experiments, a Y-tube olfactometer was used, and aphids that did not choose either of the two arms of the olfactometer (chemical or solvent) were not included in the analysis. The repellency (R) of each compound was calculated by the formula *R* = (C-T)/(C+T), where T indicates the number of aphids in the arm with the compound to be tested, and C indicates the number of aphids in the control arm. A value of *R* = 1 indicates that all the insects that have chosen were found in the control arm, while *R* = 0 indicates that as the aphids were distributed equally between the two arms, the tested substance clearly had no effect. Results are shown in **Figure 6**. The aphids were repelled significantly by (-)-α-pinene, (+)-limonene and the mixture containing (E)-β-farnesene 14.2%, (-)-α-pinene 11.8%, β-pinene 74% (Francis et al., 2005), with the *R*-values of 0.40, 0.28 and 0.48, respectively. In contrast, (±)-α -pinene, β-pinene and (E)-β-farnesene alone were not repellent for *M. viciae*, with the *R*-values of 0.07, -0.05 and 0.02, respectively (**Figure 6**).

Behavioral experiments on *M. viciae* unwinged adults were performed with the main compounds identified in a cornicle droplet. Aphids were repelled significantly by (-)-α-pinene, (+)-limonene and the mixture containing (E)-β-farnesene, (-)-α-pinene and β-pinene.

## Discussion

Odorant-binding proteins are classically defined as olfactory soluble proteins (Vogt et al., 1991; Pelosi, 1994). Since OBPs are expressed in organs devoted to chemoreception, such as antennae and mouthparts, they likely play a role related to chemoreception. The fact that OBPs are expressed in sensilla whose cuticular surface allows the entry of molecules able to stimulate the olfactory and gustatory receptors located on the sensory neurons strengthens this likelihood (Diehl et al., 2003; De Biasio et al., 2015; Pelosi et al., 2017). Considering that OBPs are also expressed in several organs not related to olfactory and gustatory perception, they can conceivably perform different functions (Nomura et al., 1992; Kitabayashi et al., 1998).

In addition, the same OBP can perform different roles when expressed in different organs and tissues which is related to the general ability of OBPs to bind and transport a range of small molecules, not only those deriving from the external environment (Jacquin-Joly et al., 2001; Zhou et al., 2004; Smartt and Erickson, 2009; Strandh et al., 2009; Sun Y.L. et al., 2012; Gu et al., 2013; Ishida et al., 2013; Zhang et al., 2013, 2015; Xia et al., 2015; Pelosi et al., 2017).

Although it is now generally recognized that OBPs are involved in cellular processes other than chemoreception, the important role of OBPs in chemoreception is confirmed. These soluble proteins, by binding small hydrophobic molecules, allow their solubilization in the sensory lymph (carrier role) and at the same time the protection against degradation performed by odorant degrading enzymes (ODEs) and the increase of sensitivity toward the receptors (Gomez-Diaz et al., 2013; Chertemps et al., 2015). In this work, we focused on the ten OBPs identified as transcripts in the aphid *Megoura viciae* antennae. Since the sensilla type and morphology provides an indication about the attribution of a hypothetical functional role of the OBPs expressed therein, an integrated and multidisciplinary approach has been adopted, starting from the analysis of the antennal ultrastructure in both wingless and winged adult morphs and on the different types of sensilla, through SEM.

Two types of trichoid sensilla (I and II) have been described in *M. viciae* adults (wingless and winged) as in other aphid species (Bromley et al., 1980; Sun et al., 2013; De Biasio et al., 2015). Four type II trichoid sensilla, with a blunt tip characterized by the presence of fissure-like structures are located on the aphid antenna distal part on the 6th segment, both in wingless and winged morphs. These fissure-like structures described for the first time on the type II trichoid sensilla at the end of the processus terminalis would appear similar to those found in the pea aphid *A. pisum* on the long hair tip of the mouthparts (De Biasio et al., 2015). In *A. pisum*, the inner lymph of fissured hair like sensilla on the mouthparts was immunostained by the antibody against an *ApisOBP (ApisOBP8).* Similarly, in *M. viciae* lymph of fissured type II trichoid sensilla on the antenna tip is immunostained by antibodies against *MvicOBP3, MvicOBP6, MvicOBP7*. The immunolocalization of all these OBPs and the simultaneous presence of fissure-like structures suggest that fissures on these types of sensilla might be involved in the entry of chemical molecules.

Otherwise, in *M. viciae*, type II trichoid sensilla located along the processus terminalis and type I trichoid sensilla visible along the whole length of the antennae are characterized by the presence of apical and longitudinal grooves similar to those observed in other insect species (Diehl et al., 2003; Palma et al., 2013; Missbach et al., 2014) where these sensilla were described as olfactory sensilla. They are morphologically different from the same category of sensilla observed in the two aphid species, *A. pisum* (De Biasio et al., 2015) and *M. persicae* (Sun et al., 2013), where a smooth surface and a rounded tip have been described. It is interesting to observe that type I trichoid sensilla in *M. viciae* are stained by antibodies against *MvicOBP1, MvicOBP3, MvicOBP6, MvicOBP7*, which is in contrast to *A. pisum* and *M. persicae* in which type I trichoid sensilla were not stained by any anti-OBP antibody, and for which a mechanoreceptive function was hypothesized (Shambaugh et al., 1978; Bromley et al., 1979; Sun et al., 2013; De Biasio et al., 2015). A possible role of *M. viciae* type I and type II trichoid sensilla in chemical perception could be hypothesized on the basis of immunolocalization signals and on the basis of the observed morphology that at the ultrastructural level highlights the presence of grooves.

Moreover, SEM observations show the presence of a single large placoid sensillum, two smaller placoid sensilla and four coeloconic sensilla (type I and II) located on the 6th segment, and a single large placoid sensillum on the 5th segment of both wingless and winged adults, as already described for *A. pisum* and for other species of aphids (Shambaugh et al., 1978; Bromley et al., 1979; Sun et al., 2013; De Biasio et al., 2015). Already available data describing the ultrastructure of aphid placoid sensilla show the localization of pore structures on these sensilla surface (Bromley et al., 1979; Sun et al., 2013). Pore like structures have been observed also in *Megoura viciae* placoid sensilla indicating that they could be typical chemosensilla as demonstrated in other aphids (Wohlers and Tjallingii, 1983; Park and Hardie, 2004). In primary rhinaria (5th and 6th segments) differences between wingless and winged adults concerning shape, distribution and number of placoid sensilla have not been observed. Secondary rhinaria on the 3rd antennal segment in *M. viciae* are constituted by placoid sensilla too, similar in the general morphology to those found in the 5th and the 6th segments, suggesting a shared chemosensory function. In winged *M. viciae* morph, about 60 placoid sensilla on the 3rd segment have been counted, whereas about 30 placoid sensilla have been counted in wingless insects on the same segment. In addition, the length of the 3rd segment increases by about 40% in winged adults. These differences suggest a potential involvement of these sensilla in the location of new host plants. Indeed, aphids acquire wings only when they need to change host plant or mate; therefore, these sensilla could be involved in the detection of plant volatiles (Pickett et al., 1992; Sun et al., 2013).

*MvicOBP1, MvicOBP3, MvicOBP6, MvicOBP7* have been immunolocalized in the lymph of placoid sensilla on the 3rd and 5th segments and in large placoid sensilla on the 6th aphid antennal segment. RT-qPCR data confirm the immunolocalization signals of *MvicOBP1* showing that the relative expression of this OBP is significantly higher in the antennae. The immunolocalization pattern of *MvicOBP6* follows what had been already described in *A. pisum* in which OBP6 was immunolocalized in placoid sensilla (large and small) on the 6th segment, in placoid sensilla on the 5th segment and in secondary rhinaria (De Biasio et al., 2015). RT-qPCR data confirm the immunolocalization signals, showing that the relative expression of *MvicOBP6* is significantly higher in the antennae. EAG experiments performed on different aphid species demonstrate that primary rhinaria (both proximal and distal) are able to perceive a range of plant volatiles. More specifically, the distal primary rhinaria (DPR) are significantly more responsive to tested alcohols than aldehydes in comparison to the proximal primary rhinaria (PPR) and vice versa, indicating a difference in the perception of plant volatiles between the two primary rhinaria (Pickett et al., 1992; van Giessen et al., 1994). Behavioral and electrophysiological studies demonstrated that secondary rhinaria in *M. viciae* and in other aphids are responsive to sex pheromone (Pettersson, 1971; Marsh, 1975; Dawson et al., 1987, 1988; Campbell et al., 1990). The immunolocalization signals of *MvicOBP1* and *MvicOBP6* both in primary and secondary rhinaria and the significantly high relative expression level of these OBPs in the antennae suggest a possible involvement of *MvicOBP1* and *MvicOBP6* in the perception of host plant chemical volatiles and sex pheromones.

RT-qPCR data also confirm the immunolocalization signals of *MvicOBP3 and MvicOBP7* showing that the relative expression of these OBPs is higher in the antennae. *A. pisum* and *M. persicae* OBP3 and OBP7, orthologs of *M. viciae* (Zhou et al., 2010), have high binding affinity to the (E)-ß-farnesene (EBF) which is the only component of the alarm pheromone in these two aphid species. The alarm pheromone triggers physiological and behavioral responses in the aphid colony, to stimulate conspecifics to leave the host plant immediately (Sun Y.F. et al., 2012). In *M. persicae* and *A. pisum*, OBP7 was immunolocalized in the primary rhinaria of the 5th and the 6th segments (PPR and DPR), but only in *M. persicae* OBP7 was also localized in the secondary rhinaria of the 3rd segment. *M. persicae* OBP3, on the other hand, was immunolocalized in the PPR and only low signals were detected in the other placoid sensilla (Sun et al., 2013) whereas *ApisOBP3* was exclusively expressed in the DPR (De Biasio et al., 2015). It had been demonstrated that in *A. pisum* the perception of EBF involves only primary rhinaria and more specifically DPR, totally excluding secondary rhinaria. Similarly, in the vetch aphid *Megoura viciae*, EBF is exclusively perceived by DPR (Wohlers and Tjallingii, 1983). Nevertheless, *MvicOBP3* and *MvicOBP7* have been immunolocalized both in primary (distal and proximal) and secondary rhinaria, unlike *ApisOBP3* and *ApisOBP7*. This may seem surprising but it is conceivable that the involvement of at least *MvicOBP3* in the perception of the other components of the alarm pheromone, as previously demonstrated (Northey et al., 2016), may take place in sensilla different from primary rhinaria. Indeed, it has been demonstrated that different OBPs can bind the same molecules in a single organism (Sun Y.F. et al., 2012). Likewise, orthologous OBPs can bind the same molecules in different organisms (Sun Y.F. et al., 2012; Northey et al., 2016) but also different molecules in different organisms (Northey et al., 2016).

Immunolocalization experiments localize *MvicOBP3* also in cornicles and cauda, which is confirmed at the mRNA level by RT-qPCR results. This finding does not represent an absolute novelty, since OBP3 expression in *A. pisum*, evaluated by RT-qPCR and immunolocalization, was also observed in cornicles and cauda (De Biasio et al., 2015). The authors hypothesized that *ApisOBP3* could be involved in the transport of the alarm pheromone EBF to the environment. Indeed, aphid cornicles are involved in the release of liquid substances in response to dangerous situations such as the presence of predators or parasitoids (Capinera, 2008). The fluid is composed of the alarm pheromone and of other lipid compounds, such as triglycerides, with sticky properties able to trap natural enemies (Strong, 1967; Callow et al., 1973; Greenway and Griffiths, 1973; Butler and O’Neil, 2006; van Emden and Harrington, 2007; De Biasio et al., 2015). Since it has been demonstrated that *MvicOBP3* binds EBF and other components of the alarm pheromone mixture (Northey et al., 2016), it is reasonable to suppose that *MvicOBP3*, expressed in the cornicles, on which hole-like structures are evident, could be involved in the transport of the alarm pheromone mixture to the environment, suggesting also in this species that OBPs could perform roles other than chemoreception.

The alarm pheromone covers an important physiological role in aphids and its use has been proposed in the development of potential strategies for aphid population control (Sun et al., 2011). The identification of OBPs able to bind this pheromone with high affinity is therefore particularly relevant. Although in most aphid species, including *A. pisum*, the major component of alarm pheromone is the EBF, in *M. viciae* the alarm pheromone is composed by a mixture of different compounds, including EBF (Bowers et al., 1972; Edwards et al., 1973; Pickett and Griffiths, 1980; Francis et al., 2005). It was demonstrated that *ApisOBP3, ApisOBP7* and ortholog proteins have high binding affinity for EBF (Sun Y.F. et al., 2012; Zhang et al., 2017). *MvicOBP3* binds EBF with high affinity but it was not able to bind the other components of the alarm pheromone ((-)-α-pinene, β-pinene, (+)-limonene) with the same affinity (Northey et al., 2016). The evaluation of the contribution of each component and the mixture to aphids repulsion behavior is required to address the identification of *MvicOBPs* binding these components. As expected, the mix of (E)-β–farnesene, (-)-α-pinene, β-pinene and (+)-limonene is significantly more repellent in comparison to the effect of the single components. Surprisingly, (E)-β–farnesene alone, as well as β-pinene alone and the racemic mixture (±)-α-pinene, is not active against *M. viciae*. The most active single components are (-)-α-pinene and (+)-limonene. The behavioral assay represents the basis to address the identification and functional characterization of *MvicOBPs* directly involved in mediating *M. viciae* dispersion behavior.

*MvicOBP8* is expressed in cornicles and in cauda long sensilla, where pores and fissure like structures have been observed, as well as in finger–like extensions that cover the entire cauda surface, different to what has been described for *A. pisum* (De Biasio et al., 2015). RT-qPCR data confirm the immunolocalization of *MvicOBP8*, showing that this OBP is significantly expressed in cornicles and cauda. It is interesting to note that, similarly to *A. pisum* OBPs, also *M. viciae* OBPs, such as *MvicOBP8* in this case, are expressed in organs apparently not related to chemoreception, such as the finger–like extensions on the cauda, suggesting a possible new function that needs to be further investigated.

In insects in general and in aphids in particular, other organs besides the antennae are related to chemoreception. SEM revealed that both the wingless and the winged vetch aphid present sensilla associated with mouthparts. Immunolocalization experiments performed on the mouthparts show that the lymph of these sensilla are stained with *MvicOBP6, MvicOBP*7 and *MvicOBP8* antibodies. RT-qPCR data confirm the immunolocalization signals of these OBPs, showing also that the relative expression levels are significantly higher in heads. In accordance with what had been already observed in *A. pisum*, whose OBP8 was immunolocalized in the sensilla on the mouthparts (De Biasio et al., 2015), *MvicOBP8* is immunolocalized in the long sensilla located on the lateral part of the labium. However, unlike what had been observed in *A. pisum*, OBP6 and OBP7 in *Megoura viciae* are found in the long hair sensilla. The observed expression patterns suggest that the three OBPs could cover a task in gustatory perception. Indeed, plant volatiles and non-volatiles (such as alkaloids and terpenoids) are moderately soluble in water and the three OBPs may be involved also in the interaction with hydrophobic non-volatile molecules (Galindo and Smith, 2001; Jeong et al., 2013; Swarup et al., 2014), suggesting a greater complexity in the mechanisms of chemoreception also involving *M. viciae* mouthparts.

Numerous trichoid sensilla have been found on the whole surface of the leg. These types of sensilla are uniform in size, shape and distribution and are similar to those already described in *A. pisum* (De Biasio et al., 2015). RT-qPCR shows a very low expression level for all the analyzed OBPs, with the exception of *MvicOBP5*, and no signal in the immunolocalization experiments.

All the results obtained by RT-qPCR experiments on the OBPs whose antibodies were already available are consistent with the results obtained by immunolocalization. We have thus carried out RT-qPCR experiments also on the other OBPs identified in the transcriptome (*MvicOBP2, MvicOBP4, MvicOBP5, MvicOBP9, MvicOBP10*), for which immunolocalization experiments have not been possible since no specific antibodies were available. All OBPs show significantly higher relative expression levels in the antennae compared to the other organs tested, allowing to hypothesize a possible role in chemoreception for these OBPs. *MvicOBP2* and *MvicOBP5* show a similar expression pattern, except for the higher relative expression level of *MvicOBP5* in legs. *MvicOBP5* is the only OBP among those identified in the transcriptome that is significantly expressed in the legs. Since in this aphid species the sex pheromone is produced and released at numerous plaques localized on the hind tarsi, a potential role for *MvicOBP5* in sex pheromone release and/or interaction can be speculated. Different roles were attributed to these organs on hind tarsi and it was suggested that they produce a sex pheromone able to attract male aphids (Flogejl, 1905; Weber, 1935; Smith, 1936; Bodenheimer and Swirski, 1957; Stroyan, 1958; Pettersson, 1971; Marsh, 1972, 1975). *MvicOBP9* show a relative expression pattern similar to *MvicOBP6* and *MvicOBP7*. Although it was not possible to evaluate the immunolocalization for this OBP, the similar expression profile suggests an analogous function. Similarly, we hypothesize that *MvicOBP10* may be involved in a task analogous to that covered by *MvicOBP1* in the light of the very similar expression pattern.

*MvicOBP3, MvicOBP5, MvicOBP7* and *MvicOBP9* are most highly expressed in IV nymphal instar and wingless morph. The observed higher expression levels of these two OBPs could relate to a higher necessity of these later developmental stages to perceive certain compounds (Roitberg and Meyers, 1978) when compared to lower transcript levels in the early stages. *MvicOBP1* displays the highest expression levels in IV nymphal instar and winged adults while *MvicOBP2, MvicOBP8* and *MvicOBP10* are primarily expressed in the winged morph. Moreover, *MvicOBP6* is mostly expressed in the first nymphal instars while *MvicOBP4* is expressed in the first nymphal instars and in the more mature instars (including the winged morph). The marked heterogeneity of our *M. viciae* OBPs expression level analysis at different developmental stages could be explained with the complexity of the molecular mechanisms that drive the behavioral response of the different aphids’ nymphal instars to the chemical molecules. Indeed, different plant chemicals are able to trigger different behavioral responses that are also dependent on aphid morph and developmental stage; moreover, different morphs of the same aphid species show different behaviors in response to the same volatiles (Lilley and Hardie, 1996; Quiroz and Niemeyer, 1998; Powell and Hardie, 2001; Webster, 2012). Within the same morph, the response to volatile compounds can vary widely in relation to the stage of development (Glinwood and Pettersson, 2000a,b).

## Conclusion

In this work we have verified which of the identified OBPs were expressed in sensilla that, for their position in typical chemoreceptive organs and for the presence of morphological features such as pores, grooves and fissure-like structures, could potentially cover chemoreceptive functions. Considering the traditional role attributed to OBPs, the gained information would have led us to assign automatically a specific role of odorants carrier toward the olfactory receptors to the identified OBPs. In the light of recent works (e.g., Larter et al., 2016) the OBPs expressed in chemosensilla are certainly involved in chemoreception but their roles can be multiple, although the specific feature of binding proteins remains unaltered (Pelosi et al., 2017). Our data on the ultrastructure of sensilla as well as on OBP expression profiles in different developmental stages and various body parts allow to state that OBPs in *Megoura viciae* show a very complex expression pattern. The increasing knowledge about the different tasks performed by OBPs in insects leads us to hypothesize that the described level of complexity of *Megoura viciae* OBPs pattern can be ascribed to the different functions of these proteins in physiological pathways of the vetch aphid. The knowledge acquired with this work could represent the road map for guiding future studies aimed to the detailed clarification of the role of each *M. viciae* OBP.

## Data Availability Statement

The raw data supporting the conclusions of this manuscript will be made available by the authors, without undue reservation, to any qualified researcher.

## Author Contributions

PF designed the experiments, and wrote and critically revised the paper. EG-W, HV, BH, J-JZ, AS, GG, RS, and SB contributed to the data interpretation and critically revised the paper. AG and DB performed the SEM experiments and immunolocalization experiments. GG, DF, and RS performed the samples collection, RT-qPCR, and antibodies validation. GG and AS performed the behavioral assays. J-JZ performed the antennal transcriptome sequencing. GG, HV, and EG-W performed the transcriptome analysis. All authors read and approved the manuscript.

## Conflict of Interest Statement

The authors declare that the research was conducted in the absence of any commercial or financial relationships that could be construed as a potential conflict of interest.

## Footnotes

1 www.graphpad.com

## Supplementary Material

FIGURE S1 Western blot performed with antisera against OBPs 1, 3, 6, 7, and 8 of *A. pisum* on protein extract from the whole *M. viciae* body (20 μg of proteins per each lane).

FIGURE S2 Alignment of amino acid sequences of *Megoura viciae* and *Acyrthosiphon pisum* OBPs

FIGURE S3 SEM images showing the distribution of sensilla on *M. viciae* legs. Trichoid sensilla present a typical hair shape and are covered by a thin cuticle (arrowheads in **(A–C)**). These sensilla show a peak with a rounded shape, without pores. Bar in **(A)**, 100 μm; bar in **(B)**, 25 μm; bar in **(C)**, 100 μm; bar in **(D)**, 2 μm

FIGURE S4 SEM images showing the distribution and morphology of different sensilla on winged *M. viciae* antennae. **(A,B)** Type II trichoid sensilla located on the terminal part of the antenna (arrowhead in **(A)**) and on processus terminalis **(B)** with grooves on tip surface (arrowhead in **(A’,B’)**). **(C)** Global view of primary rhinaria on 6th segment (arrowhead) and type II trichoid sensilla (arrow). **(D)** Details of small placoid sensilla (SP), and type I (CI) and type II (CII) coeloconic sensilla in the 6th segment surrounded by cuticular fringes (arrowheads). **(E)** Detail of porous structure on the surface of the large placoid sensillum (arrowheads). **(F)** Details of placoid sensillum of 5th segment and type I trichoid sensilla (arrow) with grooved surface (arrow in **(F’)**). Porous structures were visible on the flat surface on the placoid sensillum of this segment (arrowhead). **(G,H)** Placoid sensilla (secondary rhinaria) on the 3rd segment (white arrowhead in **(G,H)**) and trichoid sensilla type I (arrow in **(G,H)**). Bars in **(A,C,F)**, 10 μm; bars in **(A’,B’,F’)**, 500 nm; bar in **(B)**, 2 μm; bars in **(D,E)**, 2 μm; bar in **(G)**, 100 μm; bar in **(H)**, 20 μm.

FIGURE S5 Alignment of amino acid sequences of candidate OBPs from *Megoura viciae.*

FIGURE S6 *RPS9* and *RPL32* constant expression level in *M. viciae* body parts.

FIGURE S7 Relative expression level of *M. viciae* OBPs in different body parts **(A,B)** and in different nymphal instars **(C,D)** calibrated on *RPL32* and *RPS9*, respectively. OBP expression levels were quantified by RT-qPCR. Bars represent the standard deviation of the mean for 3 independent experiments. Significant differences are denoted by asterisks (Tukey’s test, (^∗^*p* < 0.05, ^∗∗^*p* < 0.01, ^∗∗∗^*p* < 0.001)). **(A,B)** Lg, legs; Cd, cornicles-cauda; Hd, head; Bd, body; An, antennae. Calibrator sample: antennae. **(C,D)** I, 1st nymphal instar; II, 2nd nymphal instar; III, 3rd nymphal instar; IV, 4th nymphal instar; Ap, apterous adults; Al, alata adults. Calibrator sample: 1st nymphal instar.

FIGURE S8 Whole-mount immunolocalization experiments showing the absence of signal for *MvicOBP8* in antenna **(A)**, *MvicOBP1* and *MvicOBP3* in the mouthparts **(C,F)**, *MvicOBP1, MvicOBP6, MvicOBP7* in the cauda **(D,G,I)**, and in cornicles **(E,H,J)**. **(B)** Negative control in which the primary antibodies were omitted. Bars in **(A,B)**, 30 μm; bars in **(C,F)**, 10 μm; bars in **(D,G,I)**, 50 μm; bars in **(E,H,J)**, 20 μm.
