# Supplementary figures and images for: Sensilla Morphology and Complex Expression Pattern of Odorant Binding Proteins in the Vetch Aphid Megoura viciae (Hemiptera: Aphididae)
Source: Front Physiol. 2018 Jun 25;9:777. doi: 10.3389/fphys.2018.00777 (PMC6027062; doi:10.3389/fphys.2018.00777)

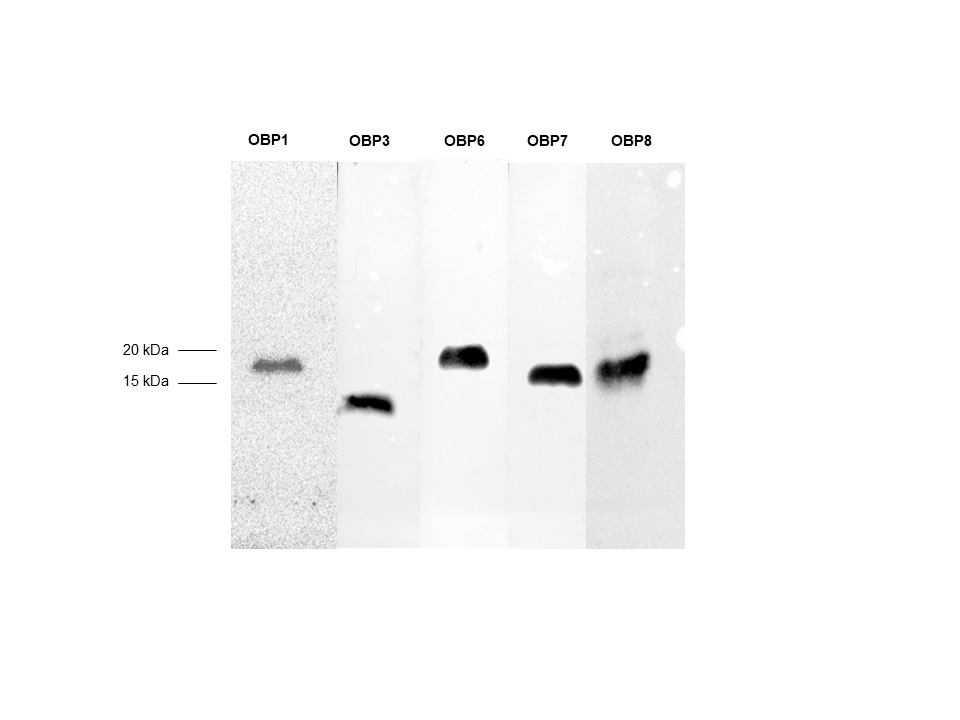

Supplement: FIGURE S1 — Western blot performed with antisera against OBPs 1, 3, 6, 7, and 8 of A. pisum on protein extract from the whole M. viciae body (20 μg of proteins per each lane). [file Image_1.TIF]

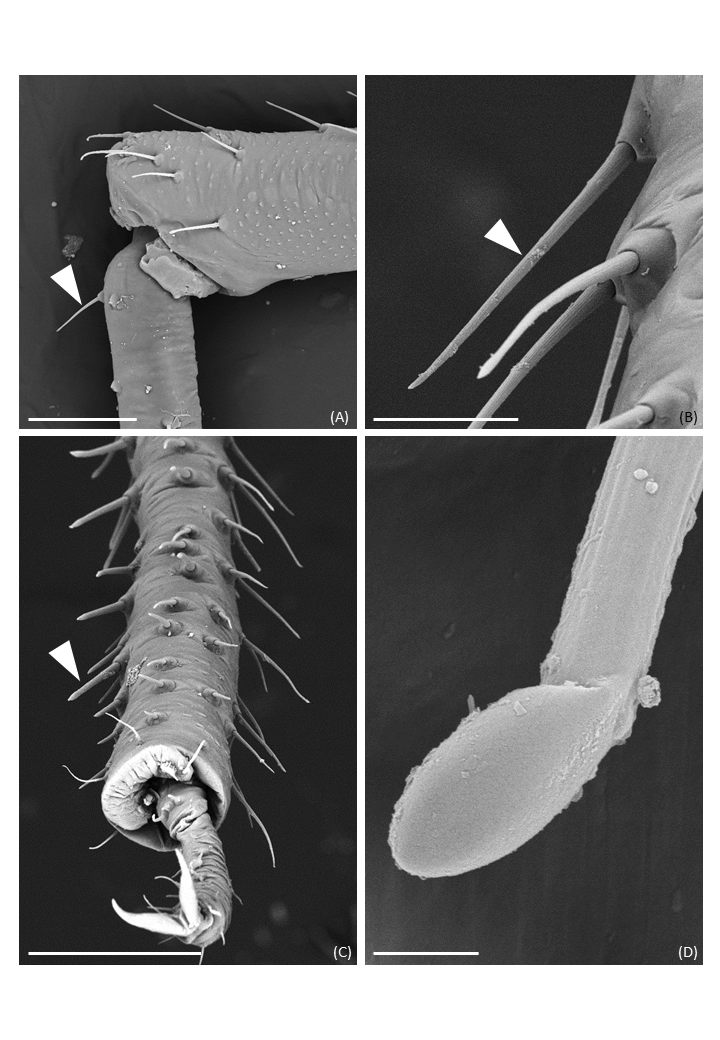

Supplement: FIGURE S3 — SEM images showing the distribution of sensilla on M. viciae legs. Trichoid sensilla present a typical hair shape and are covered by a thin cuticle (arrowheads in (A–C)). These sensilla show a peak with a rounded shape, without pores. Bar in (A), 100 μm; bar in (B), 25 μm; bar in (C), 100 μm; bar in (D), 2 μm [file Image_3.TIF]

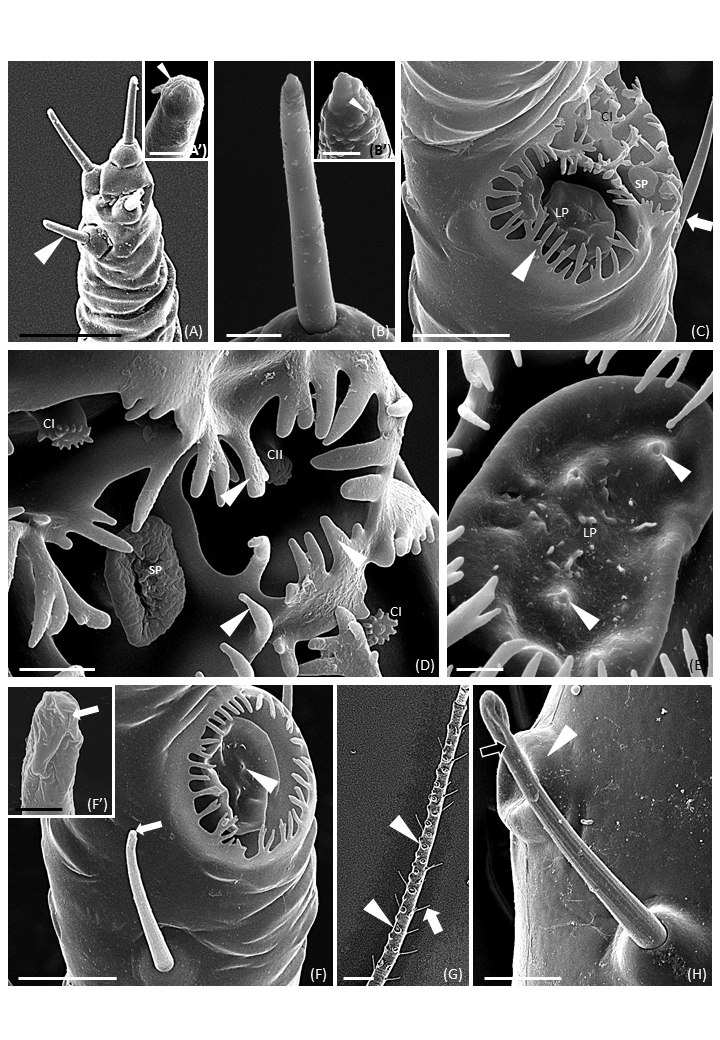

Supplement: FIGURE S4 — SEM images showing the distribution and morphology of different sensilla on winged M. viciae antennae. (A,B) Type II trichoid sensilla located on the terminal part of the antenna (arrowhead in (A)) and on processus terminalis (B) with grooves on tip surface (arrowhead in (A’,B’)). (C) Global view of primary rhinaria on 6th segment (arrowhead) and type II trichoid sensilla (arrow). (D) Details of small placoid sensilla (SP), and type I (CI) and type II (CII) coeloconic sensilla in the 6th segment surrounded by cuticular fringes (arrowheads). (E) Detail of porous structure on the surface of the large placoid sensillum (arrowheads). (F) Details of placoid sensillum of 5th segment and type I trichoid sensilla (arrow) with grooved surface (arrow in (F’)). Porous structures were visible on the flat surface on the placoid sensillum of this segment (arrowhead). (G,H) Placoid sensilla (secondary rhinaria) on the 3rd segment (white arrowhead in (G,H)) and trichoid sensilla type I (arrow in (G,H)). Bars in (A,C,F), 10 μm; bars in (A’,B’,F’), 500 nm; bar in (B), 2 μm; bars in (D,E), 2 μm; bar in (G), 100 μm; bar in (H), 20 μm. [file Image_4.TIF]

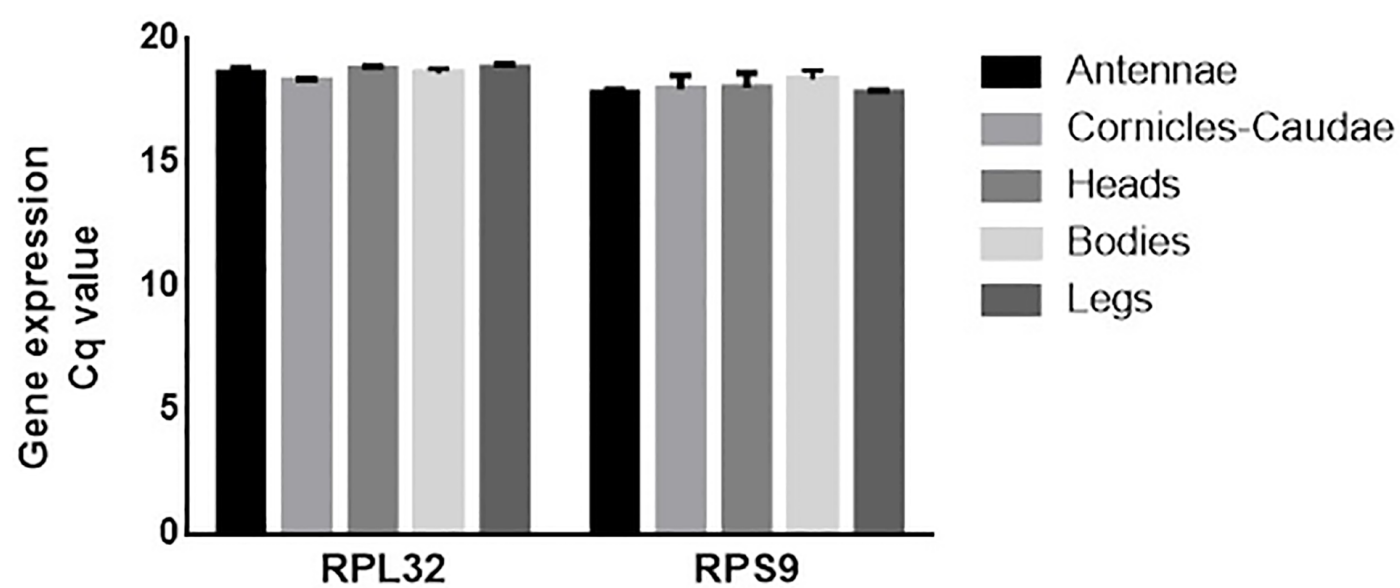

Supplement: FIGURE S6 — RPS9 and RPL32 constant expression level in M. viciae body parts. [file Image_6.PDF]

A

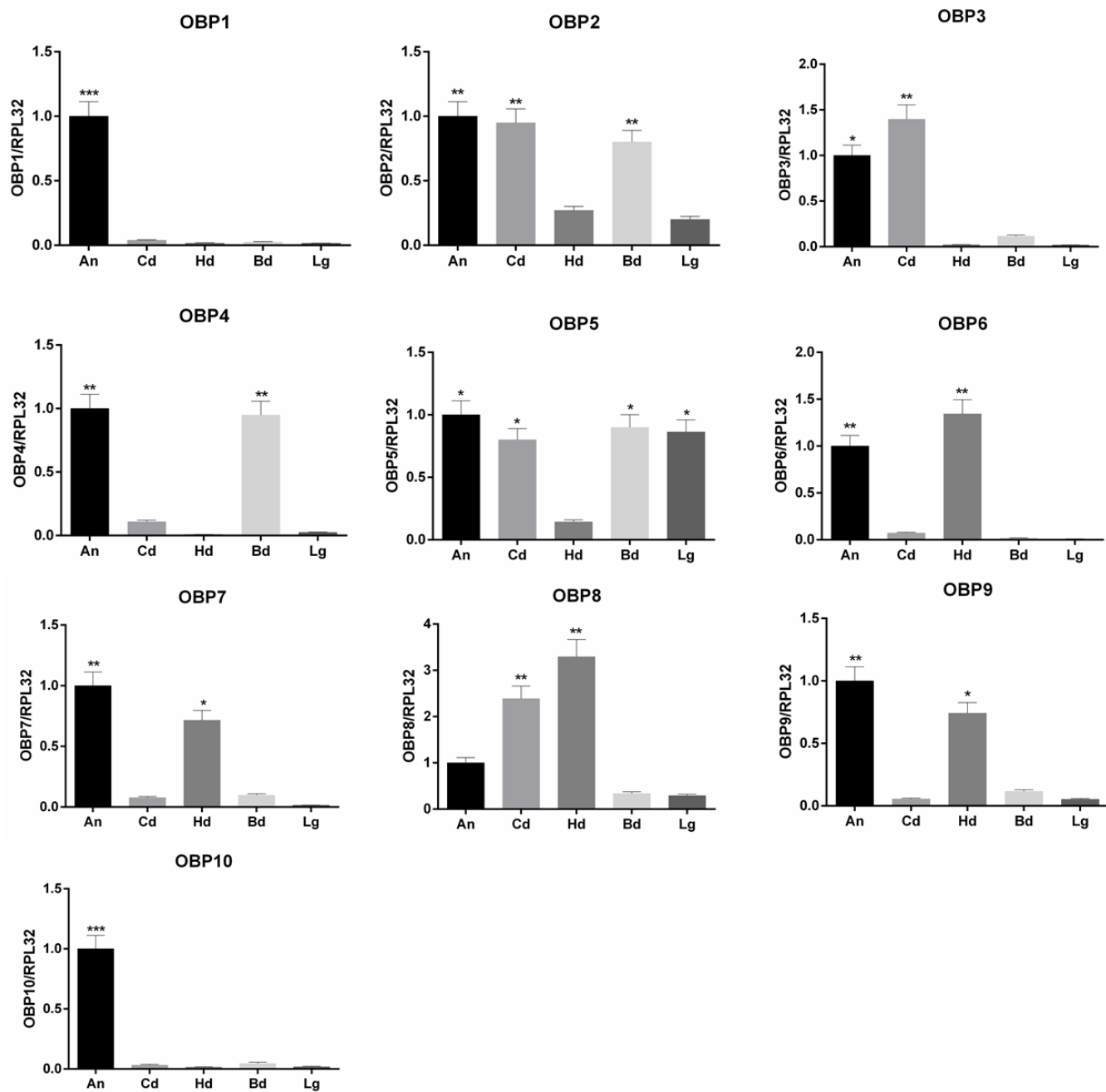

B

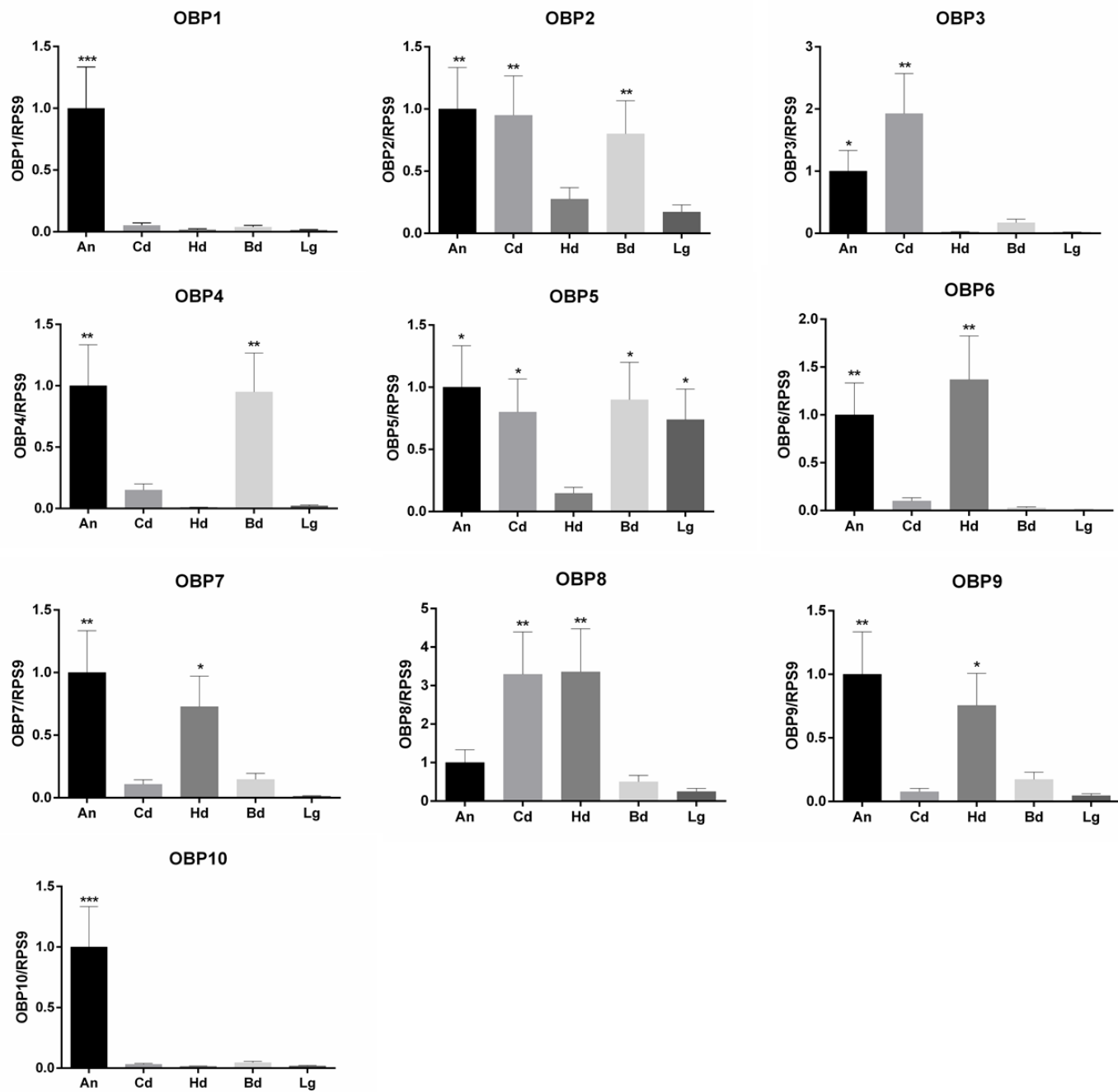

C

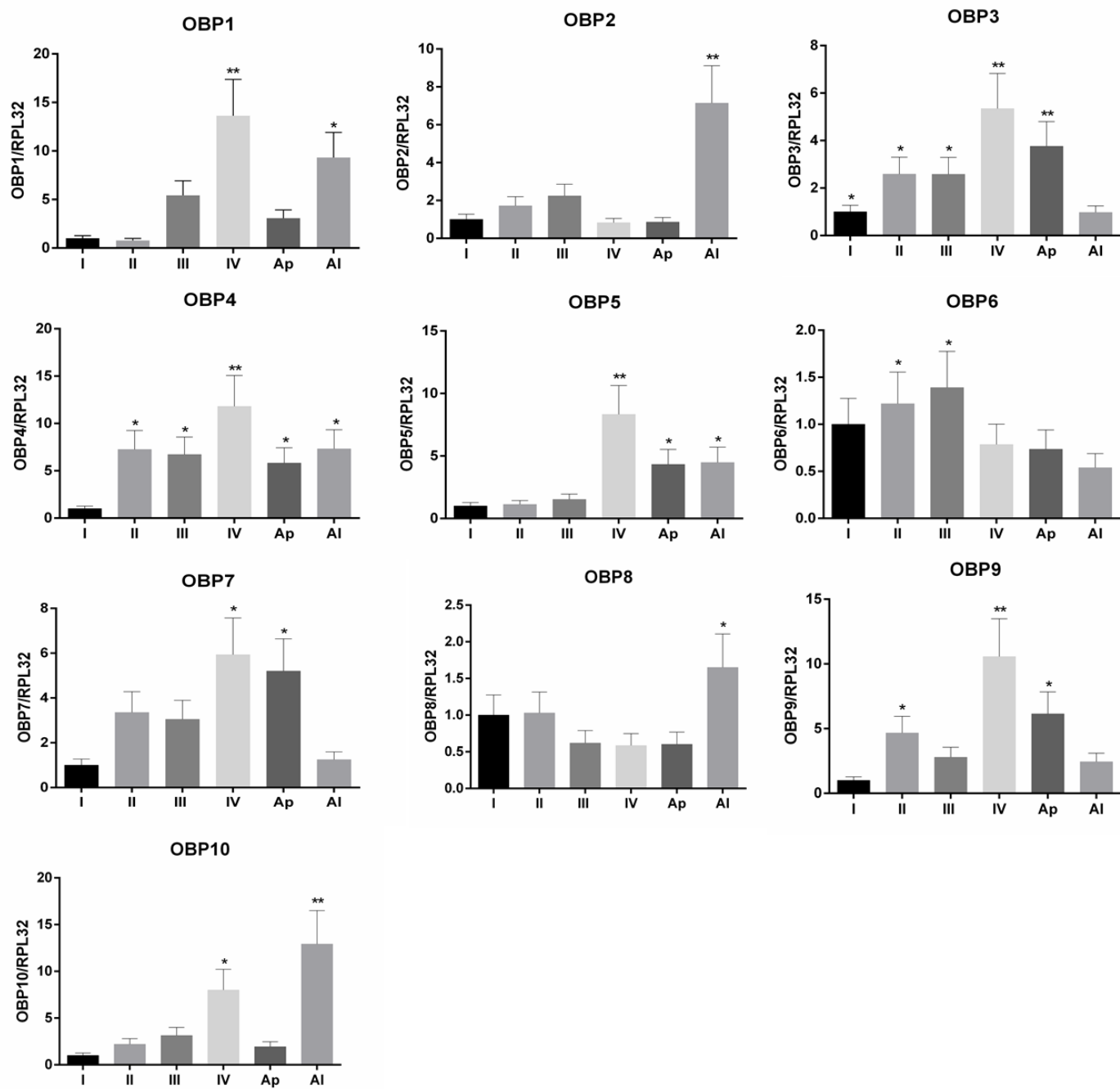

D

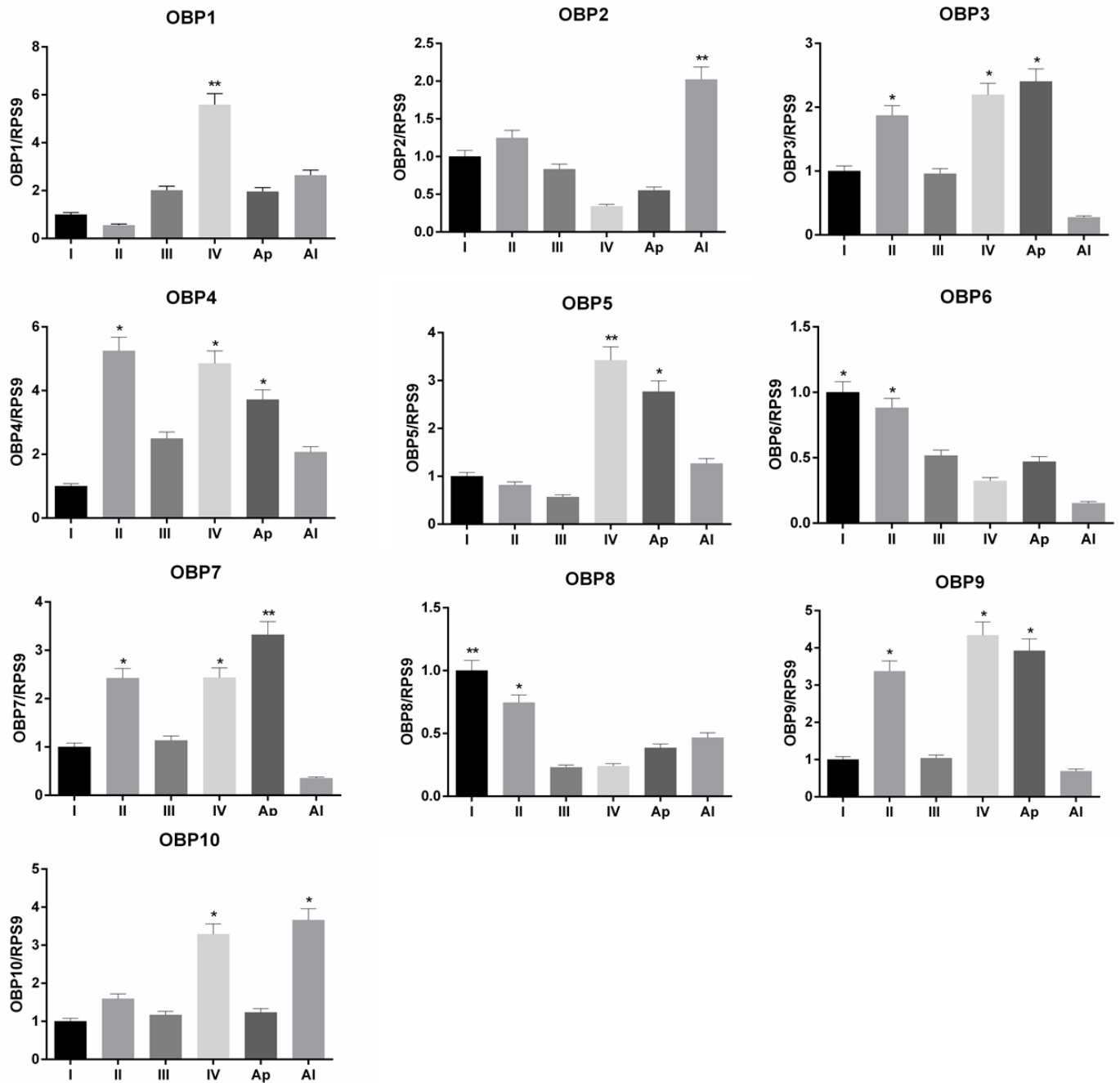

Supplement: FIGURE S7 — Relative expression level of M. viciae OBPs in different body parts (A,B) and in different nymphal instars (C,D) calibrated on RPL32 and RPS9, respectively. OBP expression levels were quantified by RT-qPCR. Bars represent the standard deviation of the mean for 3 independent experiments. Significant differences are denoted by asterisks (Tukey’s test, (∗p < 0.05, ∗∗p < 0.01, ∗∗∗p < 0.001)). (A,B) Lg, legs; Cd, cornicles-cauda; Hd, head; Bd, body; An, antennae. Calibrator sample: antennae. (C,D) I, 1st nymphal instar; II, 2nd nymphal instar; III, 3rd nymphal instar; IV, 4th nymphal instar; Ap, apterous adults; Al, alata adults. Calibrator sample: 1st nymphal instar. [file Image_7.PDF]

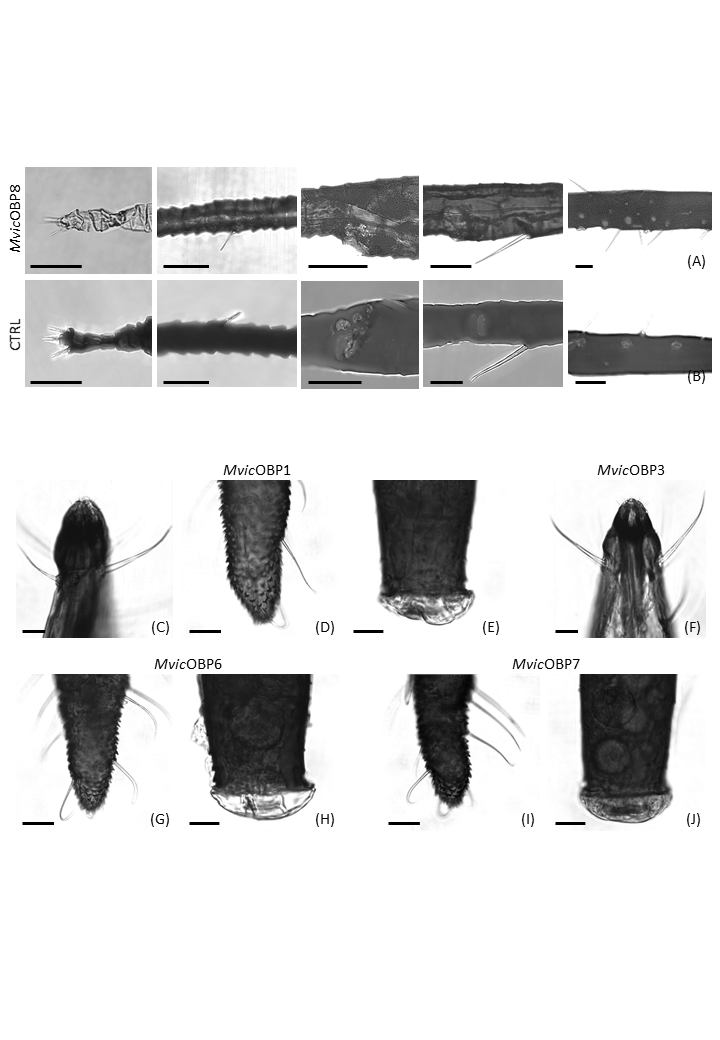

Supplement: FIGURE S8 — Whole-mount immunolocalization experiments showing the absence of signal for MvicOBP8 in antenna (A), MvicOBP1 and MvicOBP3 in the mouthparts (C,F), MvicOBP1, MvicOBP6, MvicOBP7 in the cauda (D,G,I), and in cornicles (E,H,J). (B) Negative control in which the primary antibodies were omitted. Bars in (A,B), 30 μm; bars in (C,F), 10 μm; bars in (D,G,I), 50 μm; bars in (E,H,J), 20 μm. [file Image_8.TIF]
