# Supplementary material for: Sensilla Morphology and Complex Expression Pattern of Odorant Binding Proteins in the Vetch Aphid Megoura viciae (Hemiptera: Aphididae)
Source: Front Physiol. 2018 Jun 25;9:777. doi: 10.3389/fphys.2018.00777 (PMC6027062; doi:10.3389/fphys.2018.00777)
Supplement: FIGURE S2 — Alignment of amino acid sequences of Megoura viciae and Acyrthosiphon pisum OBPs [file Image_2.PDF]

Identity 99%

```
ApisOBP1      MLNLKVMMFLCLSVIVVYCESDQVPINSSAAVESCLLETNMTRDEFEDMLTSPNARELTI   60
MvicOBP1      MLNLKVMMFLCLSVIVVYCESDQVPMNSSAAVENCLLETNMTRDEFEDMLTSPNARELTI   60
              *************************:*******.**************************

ApisOBP1      LKSHAHKCMFGCVMRKNHIVNDGVVSKEVLSKYVLNFYGRPDYKRRLIIKDVEHIVDVCA   120
MvicOBP1      LKSHAHKCMFGCVMRKNHIVNDGVVSKEVLSKYVLNFYGRPDYKRRLIIKDVEHIVDVCA   120
              ************************************************************

ApisOBP1      KKVADESETDECELAATLVTCIVLEANKAGLVDDPARQI      159
MvicOBP1      KKVADESETDECELAATLVTCIVLEANKAGLVDDPARQI      159
              ***************************************
```

Identity 95%

```
ApisOBP2      MKVSAATAVLVALVATVQSSDPCNISTCYKSGTTKPPMAVTPTHLPVQSSSTQTSHPQTT   60
MvicOBP2      MKVSAATAVLVALVATVQSSDPCNISTCYKSGTTKPPMSVTPTRLPVQSSSTPTSHPQTT   60
              **************************************:****:******** *******

ApisOBP2      YAKDHVHGSTTTKSGVNATVTTASGASVNGTEPPAVVKSSAGVTGNSTTPKPTMTEGHVA   120
MvicOBP2      YAKDHSHGSTTTKSGANATATTASGASVNGTERPAVVKSSAGVTGNLTTPKPTMTEGHVA   120
              ***** *********.***.************ ************* *************

ApisOBP2      LKQKLNTIAVKCKDELHAPQEIMALVSNTVVPQNEQQRCYLECVYKNLNLIKNNKFSVED   180
MvicOBP2      LKQKLNTIAVKCKDELHAPQEIMALVSNTVVPQNEQQRCYLECVYKNLNLIKNNKFSVED   180
              ************************************************************

ApisOBP2      GKAMARIRFANQPEEHKKAVTIIETCEKEAVIDPKTTEKCAAGRVIRNCFVKNGEKINFF   240
MvicOBP2      GKAMAKIRFANQPDEHKKAVTIIETCEKEAVIDPKTTEKCAAGRVIRNCFVKNGEKNKFL   240
              *****:*******:****************************************** :*:

ApisOBP2      PKA        243
MvicOBP2      P--        241
```

Identity 96%

```
ApisOBP3      MISSTFYITLVFGIAMLISCGHGRFTTEQIDYYGKACNASEDDLVVVKSYKVPTTETGKC   60
MvicOBP3      MISSTFYFTLLFGIAMLISCGYGRFTTEQIDYYGKACNASEDDLVVVKSYKVPSSETGKC   60
              *******:**:**********:*******************************::*****

ApisOBP3      LMKCMITKLGLLNDDGSYNKTGMEAGLKKYWSEWSTEKIESINNKCYEEALLVSKEVVAT   120
MvicOBP3      LMKCMITKLGLLNDDGSYNKTGMEAGLKKYWSEWSTEKIESINNKCYEEALLVSKEVIAT   120
              *********************************************************:**

ApisOBP3      CNYSYTVMACLNKQLDLDKST       141
MvicOBP3      CNYSYTVMACLNKQLDLDKST       141
              *********************
```

Identity 93%

```
ApisOBP4      MRGNYSSMVFLLFAIGFQDIFCQKQEPSGKCRAPDKAPLNLEIIINTCQEEIKSALLQEA   60
MvicOBP4      MRGNYSLMVFLLLAIGSQDIYCQKQELSRKCKAPDKAPLNLEIIINICQEEIKSALLQEA   60
              ****** *****:*** ***:***** * **:************** *************

ApisOBP4      LDILNDGNVEQNTPNYSSRSKREAEEDLTNEERRVAGCLLQCVYKKVKAVDETGFPVVDG   120
MvicOBP4      LDILNEGNLEQNTPSYSSRSKREADEDLTNEERRVAGCLLQCVYKKVKAVDETGFPVVDG   120
              *****:**:*****.*********:***********************************

ApisOBP4      LMKLYNEGVQDRNYYIATLSAVRHCISIAQQLKQQQPSKSFDDGQTCDLAYEMFECVSEK   180
MvicOBP4      LMKLYNEGVQDRNYYMATLSAVRHCISIAQQLKQQQPSKSFDDGQTCDLAYEMFECVSEK   180
              ***************:********************************************

ApisOBP4      IEENCGVENKSNN------ 193
MvicOBP4      IEENCGVENKSNNLSQRQV 199
              *************
```

Identity 95%

```
ApisOBP5      MSANSATIKCIAVAAILLQISVIFADAGHHRRGKELLDTEDSDFFRCKQASRKSCCGPEN  60
MvicOBP5      MSVNSLTIKCIAAAVVLLQISVIFADAGHHRRGKELLDTEDSDFFRCKQASRKSCCGPEN  60
              **.** ******.*.:********************************************

ApisOBP5      AMKRFGDKDKVAADECYAQVAEKFATVTATTPKQDLFSAEAVKITKKKQFCLHECIGKKN  120
MvicOBP5      AMKRFGDKDKVAADECYAQVAEKFATVTATTPKQDLFSADAVKITKKKQFCLHECIGKKN  120
              ***************************************:********************

ApisOBP5      NLLTEDGSLNKTFIADYAMKSVFKEQWQKQVGQKALDKCLEETYIPWPAEDKENVCNPVY  180
MvicOBP5      HLLTEDGSLNKTFIADYAMKSVFKEQWQKPVGLKALEKCLEETYIPWPAEDKENVCNPVY  180
              :**************************** ** ***:***********************

ApisOBP5      VQFQHCLWLQYESNCPANKIKITKKCEKTRNRYRMQKSTSN     221
MvicOBP5      VQFQHCLWLQYESNCPANKIKITKKCEKTRNRYRMQKLTSN     221
              ************************************* ***
```

Identity 95%

```
ApisOBP6      MQKVVFICIFAIICQTVFTAGYDRTWILRQKRGTNDDECRTLLPSSEKKLPSCCQMPNIL  60
MvicOBP6      MQKVVFICIFAIICQTVFTAGYDRTWILRQKRMTNDDECRTLIPGPEKKLPSCCQMPNIL  60
              ******************************** *********:*. **************

ApisOBP6      PNLDSTWEKCFETFKQFKDKPETKEYKEMAHGKEPPCLFQCIFMQSGLTTSDGKLNEDAI  120
MvicOBP6      PNMDSTWEKCFETFKQFKDKPETKQYKEMAHGKEPPCLFQCIFMQSGLTTSDGKLNKDAI  120
              **:*********************:*******************************:***

ApisOBP6      TKKMSEGINNDEKWKSIWQNSLNKCFDDVKQEDKKQILIMNTPAGRLMKCFLRDMYMSCP  180
MvicOBP6      TKKMSEGINNDEKWKSTWQNSLNKCFDDVKQEDKKQIPIMNTPAGRLMKCFLRDMYMSCP  180
              **************** ************************ ******************

ApisOBP6      KNVWVESSECLNMKDLVQKCPEMPPPVFKSPPKLI  215
MvicOBP6      KNVWVESSECLNVKDLVQKCPEMPPPVFKSPPQLI  215
              ************:*******************:**
```

Identity 88%

```
ApisOBP7      MVARKRMYNMLPTTVLFAIIAATVLKDCDAYLSEAAIKKTQQMLKTVCSKKHSVEEDVFT  60
MvicOBP7      MVAQKRMYNMLPTTVLFAVIAATVLKDCDAYLSETAIKKTQQMLKSVCSKKHSVNEDVFL  60
              ***:**************:***************:**********:********:****

ApisOBP7      NIKKGIFPEDNNNIKCYFACNFKTMQLINQKGVIDKKMFKDKMSMMAPPNVYKILLPVIE  120
MvicOBP7      DIKKGIFPEDNNNIKCYFACNFKTMQLINQKGSIDKKMFRDKMSMMAPPNVFNILSPVIE  120
              :******************************** ******:***********::** ****

ApisOBP7      QCTGKDKGEELCQSSYNVIKCAHSVDPKSLEFLPL  155
MvicOBP7      QCTGIDDGKELCQSSYNVIKCAHRVNPKSLEYLPL  155
              **** *.*:************** *:*****:***
```

Identity 95%

```
ApisOBP8      MFALKVACLCLSVAVVFGENNQQNGPSDRSATIFQSCIAETKLSGDALKGFRSMSIPKTQ  60
MvicOBP8      MFALKVAYLCLSVAVVFGENNQQNS-NDRSATIFQSCISETKLSGDALKGFRSMSIPKTQ  59
              ******* ****************. .***********:*********************

ApisOBP8      AEKCMMGCLMRKVNVINKGKFSVEEATKVAQKYYGTNEAMMKKAKDLIDVCAKKAQSTTE  120
MvicOBP8      AEKCMMGCLMRKVNVINKGKFSVEEATKVAQKYYGTNETMMKKAKDLIDVCAKKAQSTTE  119
              **************************************:*********************

ApisOBP8      ECALAGIVTTCIVEEAQKAGLSGGPGSRSRRTVSPKFRRDAM    162
MvicOBP8      ECALAGIVTTCIVEEAQKAGLSGGPGSRSRRTVSPKFRRNVM    161
              ***************************************:.*
```

Identity 90%

```
ApisOBP9      MIIKKTLLLSVFVLFGCLFSINKA-DDADAKDKELMSKLFTVVFKCFKDADWGTCGEMIT  59
MvicOBP9      MIIKKTLLVSVFIIFGCLFSINKAADDADAADKELISKLFTVVFKCFKDADWGTCGEMIT  60
              ********:***::********** ***** ****:************************

ApisOBP9      TKYDITQAKYKQCTCHMACAGEELGMINASGQPEPAKFLEYVNKINNPDIKSQLQLIYDK  119
MvicOBP9      TKYDITQAKYKQCTCHMACAGEELGMINSSGQPEPAKFLEYVKRINNPDIKSQLQLVYDK  120
              ****************************:*************::************:***

ApisOBP9      CQNVKGSEKCDLAEQFAICAFKESPALKERVSTLMEMLVKMKPKSK        165
MvicOBP9      CQNVKGSEKCDLAEQFAICAFKESPALKERVATLMELLVKMKPKSK        166
              *******************************:****:*********
```

Identity 81%

```
ApisOBP10     MEHLRSTNVVFAIVMALLVVQ-SSTRPQPDEMEEIKRTLYNACAGKFPITEEIKNNAKNS 59
MvicOBP10     MEHLRKTNVVFGVVIVLLVIQKSSTRPQPDELEEIKKTLYNACAGKFPITEEVKNNAKNS 60
              *****.*****.:*:.***:* *********:****:***************:*******

ApisOBP10     IISDDPTFKCFLKCCFDEMSMIDEDGIIDGDSLKAMAPDHIKPILEQVIPSCTKNVKQDG 119
MvicOBP10     IFLDDQNFKCFLKCCLDEMSLIDDDGIIDGDSLKAMASDKIKPILEQVVPNCLKDVKQDG 120
              *: ** .********:****:**:************* *:********:*.* *:*****

ApisOBP10     CEASFEFISCGIKLNPLIVALLPL   143
MvicOBP10     CEAAFDFLSCGIKLNPLTVELLPL   144
              ***:*:*:********* * ****
```
