# Supplementary material for: Sensilla Morphology and Complex Expression Pattern of Odorant Binding Proteins in the Vetch Aphid Megoura viciae (Hemiptera: Aphididae)
Source: Front Physiol. 2018 Jun 25;9:777. doi: 10.3389/fphys.2018.00777 (PMC6027062; doi:10.3389/fphys.2018.00777)
Supplement: FIGURE S5 — Alignment of amino acid sequences of candidate OBPs from Megoura viciae. [file Image_5.PDF]

```
 MvicOBP1 ---------------------------------------------------------------------------------------MLNL-KVMMFL
 MvicOBP2 MKVSAATAVLVALVATVQSSDPCNISTCYKSGTTKPPMSVTPTRLPVQSSSTPTSHPQTTYAKDHSHGSTTTKSGANATATTASGASVNGT-ERPAVV
 MvicOBP3 ---------------------------------------------------------------------------------------MISS-TFYFTL
 MvicOBP4 ---------------------------------------------------------------------------------------MRGNYSLMVFL
 MvicOBP5 -------------------------------------------------------------------------------------MSVN---SLTIKC
 MvicOBP6 ---------------------------------------------------------------------------------MVAQKRMYNM--LPTTV
 MvicOBP7 ---------------------------------------------------------------------------------------MQ---KVVFIC
 MvicOBP8 ---------------------------------------------------------------------------------------MFAL--KVAYL
 MvicOBP9 ---------------------------------------------------------------------------------------MI----IKKTL
MvicOBP10 ---------------------------------------------------------------------------------------MEHL--RKTNV

 MvicOBP1 --CLS--VIVVY-----CE---------SD---QVPINS---SAAVESCLLETNMT--R---DEFED-M----------LTSP-----NARELTIL-K
 MvicOBP2 ---KS--SAGVT-----GNLTTPKPTMTEG---HVALKQ-KLNTIAVKCKDELHAP--Q---EI-----------------MA-----LVSNTVVP-Q
 MvicOBP3 GIAML--ISCGY-----GRFT-------TE---QIDYYG-------KAC----NAS--E---DD-----------------LV-----VVKSYKVP-S
 MvicOBP4 A--IG--SQDIY-----CQKQELSGKCKAPDKAPL-----NLEIIINICQEEIKSALLQEALDILNEGNLEQNTPSYSSRSKR-----EADEDLTNEE
 MvicOBP5 AAVVLLQISVIFADAGHHR----RGKELL-DTEDSDFFRCKQASRKSCCGPENAMKRFGDKDKVAADECYAQVAEKFATVTATTPKQDLFSADTVKIT
 MvicOBP6 AVIAA--T-VLK-----DCDAYLSETA---------IKK-TQQMLKSVCSKKHSVN--E---DVFLD----------------------IKKGIFPED
 MvicOBP7 AIICQ----TVF-TAGYDRTWILRQKRMTNDDECRTLIPGPEKKLPSCCQMPNILPNMDS----TWEKCFET----FKQFKD-KPETKQY---KEMAH
 MvicOBP8 --CLS--VAVVF-----GE---------NN---QQNSND-RSATIFQSCISETKLS--G---DALKG----------------------FRSMSIP-K
 MvicOBP9 --LVS--VFIIF-----GCLFSINKAADDADAADKELIS-KLFTVVFKCFKDADWG--T---------C----------GEMI-----TTKYDITQAK
MvicOBP10 GVVIV--LLVIQ-----KSSTRPQPDE---------LEE-IKKTLYNACAGKFPIT--E---EVKNN----------------------AKNSIFL-D

 MvicOBP1 AHKCMFGCVMRKNHIVNDGVVSKEVLSKYVLNFY-GRPDY-KRRLIIKDVEHIVDVCAKKV--------AD-ESETDECEL-AATLVTCIVLEANKA-
 MvicOBP2 QQRCYLECVYKNLNLIKNNKFSVEDGKAMAKIRFANQPD------EHKKAVTIIETCEKEA-------VID-PKTTEKCAA-GRVIRNCFVKNGEKN-
 MvicOBP3 TGKCLMKCMITKLGLLNDDGSYNKTGMEAGLKKY-------WSEWSTEKIESINNKCYEEA-------LLVSKEVIATCNY-SYTVMACLNKQLDLD-
 MvicOBP4 VAGCLLQCVYKKVKAVDETGFPVVDGLMKLYNEGVQDRNY-Y----MATL-SAVRHCISIAQQLKQQQPSKSFDDGQTCDL-AYEMFECVSEKIEENC
 MvicOBP5 KQFCLHECIGKKNHLLTEDGSLNKTFIADYAMK-----SVFKEQWQKPVGLKALEKCLEETYI---PWPAEDKEN--VCNPVYVQFQHCLWLQYESNC
 MvicOBP6 NIKCYFACNFKTMQLINQKGSIDKKMFRDKMSMM-APPN------VFNILSPVIEQCTGID------------DGKELCQS-SYNVIKCAHRVNPKSL
 MvicOBP7 EPPCLFQCIFMQSGLTTSDGKLNKDAITKKMSEGINNDEKWKSTWQNS-----LNKCFDDVKQ-------EDKKQIPIMNTPAGRLMKCFLRDMYMSC
 MvicOBP8 AEKCMMGCLMRKVNVINKGKFSVEEATKVAQKYY-GTNE-----TMMKKAKDLIDVCAKKA-----------QSTTEECAL-AGIVTTCIVEEAQKA-
 MvicOBP9 QCTCHMACAGEELGMINSSGQPEPAKFLEYVKRI-NNPD------IKSQLQLVYDKCQN-------------VKGSEKCDL-AEQFAICAFKESPALK
MvicOBP10 NFKCFLKCCLDEMSLIDDDGIIDGDSLKA----M-ASDK------IKPILEQVVPNCLK-D------------VKQDGCEA-AFDFLSCGIKLNPLTV

 MvicOBP1 VDD------PARQI--------------------
 MvicOBP2 LP--------------------------------
 MvicOBP3 T---------------------------------
 MvicOBP4 ENK------SNNLSQRQV----------------
 MvicOBP5 NKIKITKKCEKTRNRYRMQ-KL-----TSN----
 MvicOBP6 LPL-------------------------------
 MvicOBP7 NVWVESSECLNVKDLVQKCPEMPPPVFKSPPQLI
 MvicOBP8 SGG------PGSRSRRTVSPKFRRNVM-------
 MvicOBP9 VAT------LM-ELLVKMKPKSK-----------
MvicOBP10 LPL-------------------------------
```
